# Association between occupational heat exposure and early renal dysfunction among Chinese petrochemical workers: a combined machine learning and WQS modeling study

**DOI:** 10.3389/fpubh.2025.1648619

**Published:** 2025-11-12

**Authors:** Qingyu Li, Chuancheng Wu, Minhua Li, Yilin Zhang, Yifeng Chen, Shanshan Du, Rong Xu, Zihu Lv, Weimin Ye, Wei Zheng, Jianjun Xiang

**Affiliations:** 1 Department of Preventive Medicine, School of Public Health, Fujian Medical University, Fuzhou, China; 2 Key Laboratory of Environment and Health of Fujian Higher Education Institutes, School of Public Health, Fujian Medical University, Fuzhou, China; 3 Institute of Population Medicine, Fujian Medical University, Fuzhou, China; 4 Quangang Hospital, Quanzhou, China; 5 Department of Epidemiology and Health Statistics, School of Public Health, Fujian Medical University, Fuzhou, China; 6 The First Affiliated Hospital of Fujian Medical University, Fuzhou, China

**Keywords:** occupational heat exposure, hyperuricemia, petrochemical workers, machine learning, renal dysfunction

## Abstract

**Objective:**

To investigate the association between occupational heat exposure and hyperuricemia among petrochemical workers.

**Methods:**

We retrospectively analyzed the association between workplace heat exposure and hyperuricemia by using 10 years of occupational health examination records from 2,312 petrochemical workers in Fujian Province, China. Generalized linear models (GLMs) were employed to estimate the effects of individual exposures. Weighted quantile sum (WQS) regression model was used to evaluate the combined effects of multiple occupational exposures and to identify the relative contribution of each exposure factor. A hyperuricemia risk prediction model was developed using the LightGBM machine-learning algorithm, with feature importance assessed using SHAP (SHapley Additive exPlanations) values.

**Results:**

Occupational heat exposure was significantly associated with an increased risk of hyperuricemia (OR = 1.68, 95% CI: 1.28–2.20). In the GLM analysis, co-exposure to heat with benzene (OR = 1.93, 95% CI 1.05–3.55), H_2_S (OR = 3.38, 95% CI 1.94–5.88), gasoline (OR = 2.58, 95% CI 1.49–4.48), acid anhydride (OR = 2.21, 95% CI 1.09–4.48) and CO (OR = 2.14, 95% CI 1.16–3.97) further increased the risk (all *p* < 0.05), suggesting synergistic effects. The WQS analysis indicated that in the mixed occupational hazards exposure, heat exposure (49.2%) contributing nearly half the effect to the overall effect. The LightGBM machine learning model identified length of service, age, BMI, gender, and heat exposure as the main predictors of hyperuricemia. The SHAP analysis confirmed heat exposure as a key independent contributor alongside length of service.

**Conclusion:**

Occupational heat exposure in petrochemical settings is significantly associated with hyperuricemia, suggesting potential early renal dysfunction risk. Integrating machine learning–based predictive models into workplace health surveillance may facilitate the early identification and management of high-risk workers. However, causal inference remains limited by the retrospective design and potential residual confounding, underscoring the need for prospective studies to validate and extend these findings.

## Graphical abstract

**Figure:**
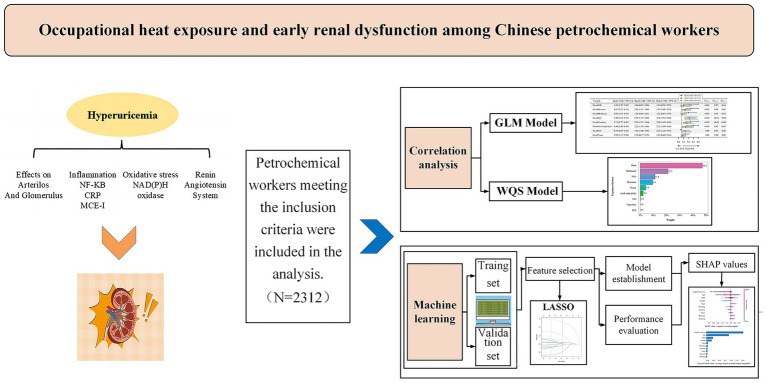

## 1 Introduction

The petrochemical industry involves the processing and transformation of petroleum-derived chemical raw materials, where high temperatures are essential for operations like catalytic cracking and hydrocracking (1, 2). Thus, occupational heat exposure represents a significant workplace hazard. In recent years, climate change-induced extreme heat events have further exacerbated the burden of workplace heat exposure (3), which may significantly increase health risks among petrochemical workers (4, 5). The kidneys, which play a vital role in maintaining fluid and electrolyte homeostasis and in excreting metabolic waste products, are particularly vulnerable to heat stress (6). Under heat stress, peripheral vasodilation and sweating lead to substantial fluid loss, resulting in dehydration and reduced urinary excretion, which in turn elevates serum uric acid levels. Moreover, heat stress induces systemic oxidative stress and inflammatory responses that further impair renal function (7), thereby contributing to the development of both acute kidney injury (AKI) and chronic kidney disease (CKD) (8).

An increasing body of evidence has linked occupational heat exposure to renal dysfunction (9–11). Archived case reports from the US Occupational Safety and Health Administration (OSHA) indicate that heat-related acute kidney injury (HR-AKI) occurs across diverse occupational settings, both indoors and outdoors (12). However, research focusing on the renal health effects of heat exposure among petrochemical workers remains limited. Our preliminary analysis identified hyperuricemia as the most common abnormality related to impaired kidney function among petrochemical workers. As the end product of purine metabolism, uric acid contributes to intracellular oxidation, endothelial dysfunction, renal fibrosis, and glomerulosclerosis (13, 14). Furthermore, hyperuricemia is an established independent risk factor for the development of incident CKD and rapid renal function decline, as well as a biomarker for early-stage renal dysfunction (15, 16), being closely associated with declining eGFR, albuminuria, and kidney failure (17). Evidence also suggests that treating hyperuricemia in its early stage may delay or even prevent the onset of CKD (18). Therefore, hyperuricemia was selected as the primary indicator of early renal dysfunction in this study. Existing epidemiological studies have reported a higher prevalence of chronic kidney disease (17.9%) among residents near refineries compared to the general population (12.3%) (19), along with elevated uric acid levels in oilfield workers and local wildlife (20, 21). These findings highlight the need to investigate the association between occupational heat exposure and hyperuricemia among petrochemical workers to better inform targeted heat-related health protection strategies.

Occupational exposures in the petrochemical industry often involve co-exposure to multiple hazards, such as benzene, H_2_S and other chemicals, which may interact synergistically and amplify health risks. Existing evidence suggests that the combination of thermal and chemical exposures in petrochemical operations contributes to increased vulnerability to a range of diseases (22, 23). Traditional single-exposure statistical models are often inadequate for capturing the complex interactions of multiple exposures. By contrast, WQS regression can generate exposure indices that identify the most influential risk factors while accounting for interaction effects among exposures (24). Furthermore, the integration of machine learning with SHAP values offers robust methodologies for investigating nonlinear and high-dimensional relationships inherent in chemical and environmental mixtures (25, 26). Therefore, by combining multiple statistical models and machine learning algorithms, this study may yield valuable insights for developing prevention strategies and promoting the occupational health of petrochemical workers. In this study, we analyzed the association between occupational heat exposure and hyperuricemia among petrochemical workers using multiple statistical models and machine learning methodologies, with aims to provide evidence-based guidance for occupational health management and mitigate the burden of heat-related renal impairment in high-risk industrial settings.

## 2 Materials and methods

### 2.1 Study design and participants

This retrospective study was conducted using occupational health examination records collected between January 2013 and December 2022 from the Quangang Petrochemical Industrial Park (QPIP), Quanzhou, Fujian Province, China. Established in 2005, QPIP covers an area of about 30 square kilometers and has a population of around 360,000. Its industrial chain mainly involves ethylene, propylene, carbon tetrachloride, benzene and paraxylene (27).

Under the Law on the Prevention and Control of Occupational Diseases, employers are required to provide regular health examinations for workers exposed to occupational hazards, typically conducted from March to August each year. This uniform schedule reduces seasonal variability between individuals. According to the examination protocol, all workers were instructed to fast overnight before the examination and to remain fasting on the morning of the test day. In this study, historical occupational health records were obtained from the Minnan Branch of the First Affiliated Hospital of Fujian Medical University. It is the sole government-designated hospital authorized to conduct occupational health examinations in the region. All data were de-identified before delivery. All raw data underwent standardized processing, including variable recoding, value assignment, labeling, and logical consistency check. To ensure accuracy and completeness, the processed dataset was further validated through consultations with occupational health experts and enterprise management personnel.

The extracted data includes the following information: (1) Demographic characteristics, such as gender, age, and lifestyle factors (including smoking/drinking frequency); (2) General physical examination, such as height, weight, and body mass index (BMI); (3) Pre-existing chronic conditions (e.g., hyperglycemia, hypertension); and (4) Occupational exposure profile, such as length of service, types of occupational hazards (e.g., heat, benzene, methanol, gasoline, acid anhydrides, carbon monoxide (CO), hydrogen sulfide (H_2_S), ammonia (NH_3_), and noise). Routine monitoring of these occupational hazard factors was conducted by Fujian Center for Prevention and Control of Occupational Disease and Chemical Poisoning, which classified and reported exposures according to the National Occupational Hazard Detection Criteria. These records were incorporated into workers’ occupational health files. The study was approved by the Medical Ethics Committee of Fujian Medical University (Approval No.: Fuyi Medical Ethics Review No. 111).

Workers were included in the analysis if they met the following criteria: (1) Aged 18–65 years with ≥1 year of continuous frontline work in petrochemical production; and (2) With complete occupational exposure documentation. The following workers were excluded from data analysis: (1) Pre-existing diagnosis of CKD; (2) Missing key occupational exposure and outcome data in health records; (3) Temporary or short-term rotational work of < 1 year; (4) Comorbid thyroid disorders, hepatic dysfunction, or pre-existing renal impairment; and (5) History of severe cardiovascular diseases or malignant tumors. Among the occupational health examination records obtained from 2013 to 2022, not all workers participated in every examination, and to avoid within-person correlation, we structured the dataset such that each worker contributed only one observation: the first record with hyperuricemia or, if none, the most recent record without hyperuricemia. Thus, the dataset did not include repeated observations from the same individual. Finally, 2,312 petrochemical workers were included in the analysis.

### 2.2 Definitions of exposure variables and health outcomes

In this study, heat exposure is a categorical hazard (variable) documented in the occupational health examination records. This classification was based on occupational monitoring data collected annually by the Fujian Center for Prevention and Control of Occupational Disease and Chemical Poisoning. The classification thresholds were defined in accordance with GBZ 2.2–2007: Measurement of physical factors in the workplace Part 7. High-temperature work is defined as an operation in which the average WBGT index at the worksite is ≥25 °C during production activities (28, 29).

Body mass index (BMI): According to cut-off points for Chinese adults, overweight and obesity were defined as 24 ≤ BMI < 28 and BMI ≥ 28 kg/m^2^, respectively (30).

Frequency of drinking was divided into three conditions: (1) Never drinking was defined as not consuming any alcohol or alcoholic beverages for at least 6 months prior to the survey; (2) Often drinking was defined as consuming alcohol at least twice a week, with an intake greater than 50 g per occasion, for more than half a year; and (3) Occasionally drinking was between often drinking and never drinking.

Smoking frequency was divided into three conditions: (1) Never smoking was defined as not smoking for at least 6 months; (2) Often smoking was defined as smoking ≥1 cigarette per day or ≥7 cigarettes per week for at least half a year within the past year; and (3) Occasional smoking is between often smoking and never smoking.

Hyperuricemia was defined as serum uric acid ≥ 420 μmol/L (7.0 mg/dL) in males and serum uric acid ≥ 360 μmol/L (6.0 mg/dL) in females (31).

Hypertension: According to The Chinese Guidelines for Prevention and Treatment of Hypertension (2024 Revision) (32), it is defined as systolic blood pressure (SBP) ≥ 140 mmHg and/or diastolic blood pressure (DBP) ≥ 90 mmHg without any antihypertensive drugs, or those who have a previous history of hypertension and are currently taking antihypertensive drugs.

Hyperglycemia: According to The Chinese Guidelines for the Prevention and Treatment of Type 2 Diabetes criteria (33), fasting blood glucose>7.0 mmol/L; glycosylated hemoglobin level>6.5%, or self-reported formal institutional diagnosis of diabetes, or currently receiving hypoglycemic drugs.

### 2.3 Statistical analysis

Descriptive analysis was conducted to examine the characteristics of occupational hazards exposure and the main occupational health issues among petrochemical workers. Continuous variables were presented as mean ± standard deviation (X̅±SD), and comparisons between groups were conducted using independent sample t-tests. Categorical data are presented as frequencies and percentages (N, %), with group comparisons conducted using the chi-square test.

Three-stage stepwise adjusted GLM models were established to investigate the associations between each occupational hazard and hyperuricemia. Model 1 (unadjusted) included the single occupational hazard factor without adjusting for any covariates. Model 2 (basic adjustment) was adjusted for gender, age, and BMI. Model 3 (fully adjusted) was further adjusted for additional confounders, including length of service, hyperglycemia, hypertension, smoking, and alcohol. Covariate selection was guided by a DAG developed from prior literature and study variables. In addition, we explored potential unmeasured confounders between occupational hazards and hyperuricemia by calculating E-value. Additional stratified analyses were conducted by gender, age, BMI, and length of service to explore potential effect modification.

To evaluate the joint effects of combined exposure to heat and other occupational exposures, GLMs were performed to estimate the multiplicative interactions. Additive interactions were assessed using the relative excess risk of interaction (RERI), the attributable proportion of interaction (AP), and the synergy index (SI). The estimated interaction effects and their 95% confidence intervals (95% CI) were visualized using forest plots.

To investigate the combined effects of mixed occupational hazards on hyperuricemia and assess the effect contributions of individual factors, a WQS regression model was constructed incorporating 9 occupational hazards (34). Given the primary variables were categorical, WQS regression is well-suited for the analysis of categorical or ordinal exposure indicators. The number of quantiles was set to null. The dataset was randomly split into training (60%) and validation (40%) subsets (random seed = 1800). Each exposure variable was assigned a weight using 200 bootstrap samplings to screen factors that contribute significantly to the outcome, constraining the overall effect direction to be positive (*β* > 0). Weight distributions were visualized using kernel density plots. To further test the robustness of the results, we conducted negative-direction analysis of WQS model and varied the train/validation split and random seed in the WQS model.

In the initial model comparison step, nine machine learning algorithms were evaluated to identify the optimal classifier. The dataset was randomly partitioned into training (70%) and validation (30%) subsets to assess the predictive performance of each model. To prevent data leakage, all preprocessing steps, including missing data imputation, feature scaling, and feature selection (collinearity diagnosis and LASSO regression) were performed strictly within each training fold during 10-fold cross-validation, random seeds:1000. Key predictors identified by LASSO were then used to train nine machine learning models: Logistic Regression (LR), XGBoost Classifier (XGB), LightGBM Classifier (LGBM), Random Forest Classifier (RF), AdaBoost Classifier (AdaBoost), Gaussian Naive Bayes (GaussianNB), Complement Naive Bayes (ComplementNB), Multilayer Perceptron Classifier (MLP), Support Vector Classifier (SVC). Model performance was evaluated using receiver operating characteristic (ROC), area under the curve (AUC), negative predictive value, precision, recall, sensitivity, F1 score, and decision curve analysis (DCA). In further developing a hyperuricemia prediction model using the LightGBM classifier, the dataset was randomly divided into training (70%) and test (30%) subsets, and the validation set was generated from the training subset through a 10-fold cross-validation procedure. Model interpretability was enhanced using SHAP values to quantify the contribution of each predictor.

All analyses were performed using R (version 4.2.3), python (version 3.11.4), and SAS (version 9.4). Statistical tests were conducted using two-sided tests, and a *p* < 0.05 was considered statistically significant.

## 3 Results

### 3.1 Comparison of characteristics between hyperuricemia and control groups

In this study, among the 2,312 workers, 1,390 (60.1%) had hyperuricemia. Compared to those without hyperuricemia, affected workers had significantly higher BMI (24.23 vs. 22.98 kg/m^2^) and a higher proportion of males (83.96% vs. 51.84%). As shown in Table 1, workers with hyperuricemia were more likely exposed to occupational hazards, including heat, NH_3_, benzene, methanol, acid anhydrides, CO, and noise (all *p* < 0.05).

**Table 1:** Characteristics of petrochemical workers between groups with or without hyperuricemia, n (%).

Variable	Total (*n* = 2,312)	Control (*n* = 922)	Hyperuricemia (*n* = 1,390)	χ^2^/ *t*	*p*-value
BMI (kg/m^2^)	23.73 ± 3.12	22.98 ± 2.92	24.23 ± 3.15	9.58	**<0.001**
Age (years)	40.74 ± 11.21	44.42 ± 11.15	38.31 ± 10.57	13.31	**<0.001**
Length of service (years)	20.05 ± 12.04	23.20 ± 12.25	17.96 ± 11.44	10.48	**<0.001**
Gender					
Female	667 (28.85)	444 (48.16)	223 (16.04)	278.49	**<0.001**
Male	1,645 (71.15)	478 (51.84)	1,167 (83.96)		
Smoking					
Never	1,602 (69.29)	714 (77.44)	888 (63.89)	60.11	**<0.001**
Occasionally	263 (11.38)	55 (5.97)	208 (14.96)		
Often	447 (19.33)	153 (16.59)	294 (21.15)		
Drinking					
Never	1,226 (53.03)	574 (62.26)	652 (46.91)	71.11	**<0.001**
Occasionally	889 (38.45)	258 (27.98)	631 (45.39)		
Often	197 (8.52)	90 (9.76)	107 (7.70)		
Heat					
No	1834 (79.33)	814 (88.29)	1,020 (73.38)	75.09	**<0.001**
Yes	478 (20.67)	108 (11.71)	370 (26.62)		
NH_3_					
No	2018 (87.28)	862 (93.49)	1,156 (83.17)	53.26	**<0.001**
Yes	294 (12.72)	60 (6.51)	234 (16.83)		
Benzene					
No	1880 (81.31)	800 (86.77)	1,080 (77.70)	30.01	**<0.001**
Yes	432 (18.69)	122 (13.23)	310 (22.30)		
Methanol					
No	1958 (84.69)	827 (89.70)	1,131 (81.37)	29.66	**<0.001**
Yes	354 (15.31)	95 (10.30)	259 (18.63)		
H_2_S					
No	1,519 (65.70)	625 (67.79)	894 (64.32)	2.96	0.085
Yes	793 (34.30)	297 (32.21)	496 (35.68)		
Gasoline					
No	1,616 (69.90)	643 (69.74)	973 (70.00)	0.02	0.894
Yes	696 (30.10)	279 (30.26)	417 (30.00)		
Acid anhydrides					
No	1969 (85.16)	832 (90.24)	1,137 (81.80)	31.25	**<0.001**
Yes	343 (14.84)	90 (9.76)	253 (18.20)		
CO					
No	1872 (80.97)	797 (86.44)	1,075 (77.34)	29.82	**<0.001**
Yes	440 (19.03)	125 (13.56)	315 (22.66)		
Noise					
No	1,351 (58.43)	571 (61.93)	780 (56.12)	7.72	**0.005**
Yes	961 (41.57)	351 (38.07)	610 (43.88)		
Hyperglycemia					
No	2,284 (98.79)	908 (98.48)	1,376 (98.99)	1.21	0.271
Yes	28 (1.21)	14 (1.52)	14 (1.01)		
Hypertension					
No	2077 (89.84)	824 (89.37)	1,253 (90.14)	0.36	0.547
Yes	235 (10.16)	98 (10.63)	137 (9.86)		

Bold cells mean statistically significant.

### 3.2 Association between occupational hazards and hyperuricemia

**Figure 1:**
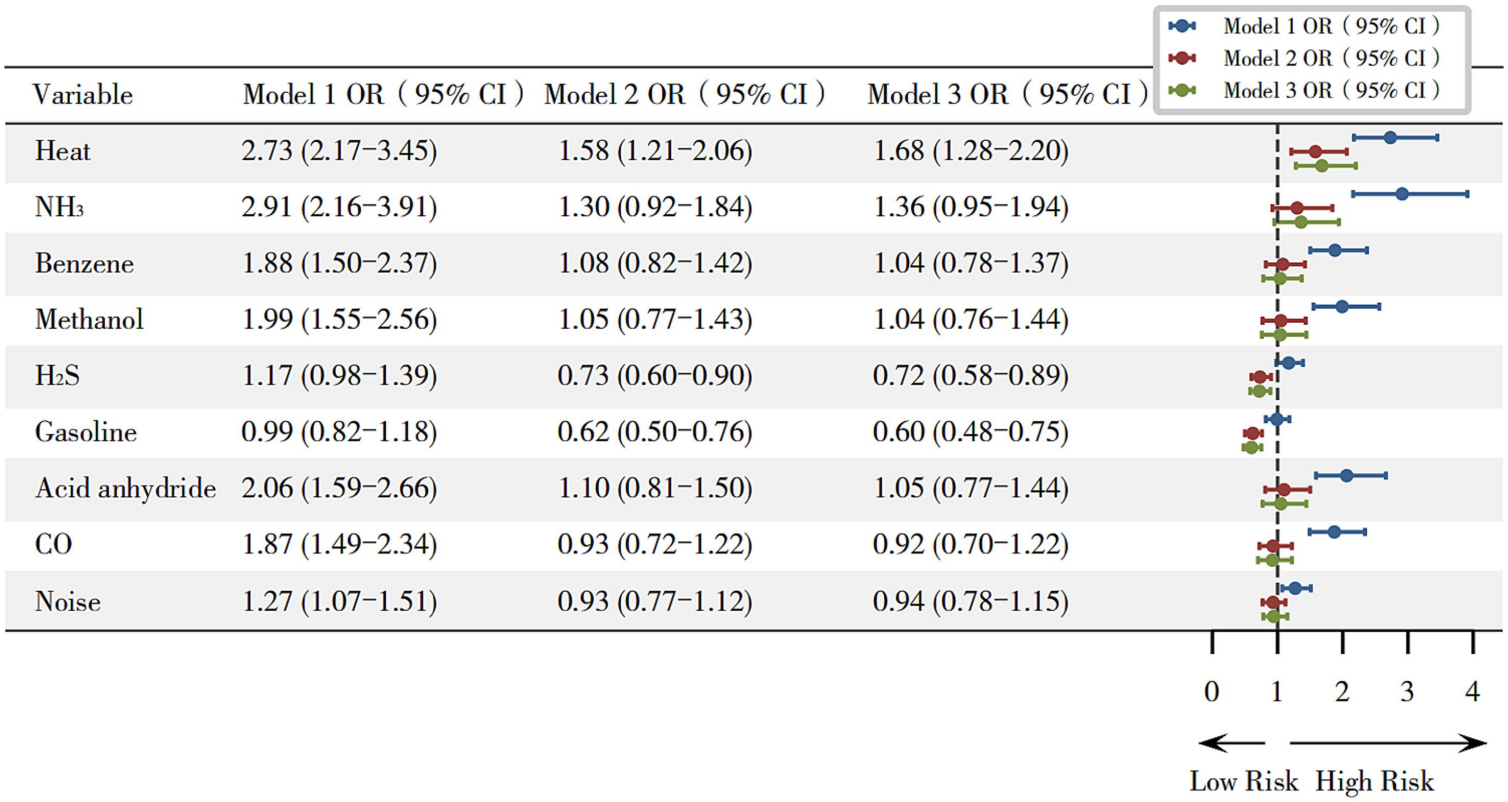
Odds ratios for hyperuricemia associated with occupational hazards. Model 1 (unadjusted); Model 2 (adjusted for gender, age, and BMI); and Model 3 (adjusted for gender, age, and BMI, length of service, hyperglycemia, hypertension, smoking, and alcohol consumption). *p < 0.05.

A three-stage stepwise GLM was used to analyze each hazard’s association with hyperuricemia. As shown in Figure 1, in unadjusted analyses, exposure to heat (OR = 2.73, 95% CI: 2.17–3.45), NH_3_ (OR = 2.91, 95% CI: 2.16–3.91), benzene (OR = 1.88, 95% CI: 1.50–2.37), methanol (OR = 1.99, 95% CI: 1.55–2.56), acid anhydrides (OR = 2.06, 95% CI: 1.59–2.66), CO (OR = 1.87, 95% CI: 1.49–2.34) and noise (OR = 1.27, 95% CI: 1.07–1.51) was associated with a higher risk of hyperuricemia (*p* < 0.05). After adjustment for age, gender, and BMI, exposure to heat (OR = 1.58, 95% CI: 1.21–2.06) remained statistically significant. In the fully adjusted model, occupational heat exposure remained a significant predictor of hyperuricemia (OR = 1.68, 95% CI: 1.28–2.20), while exposure to gasoline (OR = 0.60, 95% CI: 0.48–0.75) and H_2_S (OR = 0.72, 95% CI: 0.58–0.89) were associated with lower odds of hyperuricemia.

### 3.3 The contribution proportions of various occupational exposures to hyperuricemia

**Figure 2:**
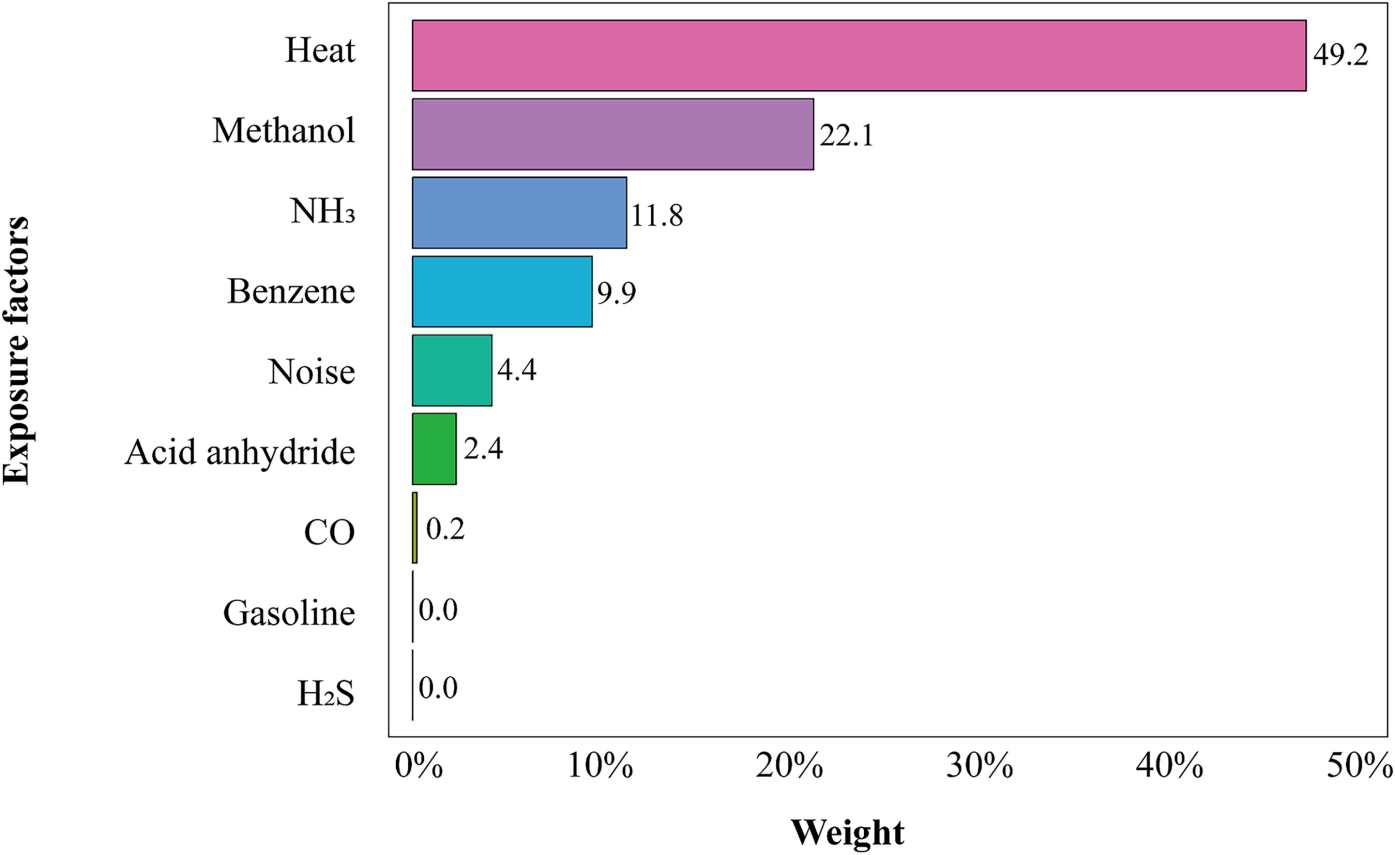
WQS regression weights for each occupational exposure. Covariates were adjusted for gender, age, length of service, smoking, alcohol consumption, and BMI. The bar heights indicate each hazard’s contribution to the hyperuricemia risk index.

Figure 2 presents the weights of each hazard in the WQS model. Occupational heat exposure had the highest weight (49.2%), indicating it was the dominant contributor to the combined risk. Methanol and NH_3_ were the next largest contributors (22.1 and 11.8%, respectively).

### 3.4 Joint effects of combined exposure to heat and other occupational hazards on hyperuricemia

**Figure 3:**
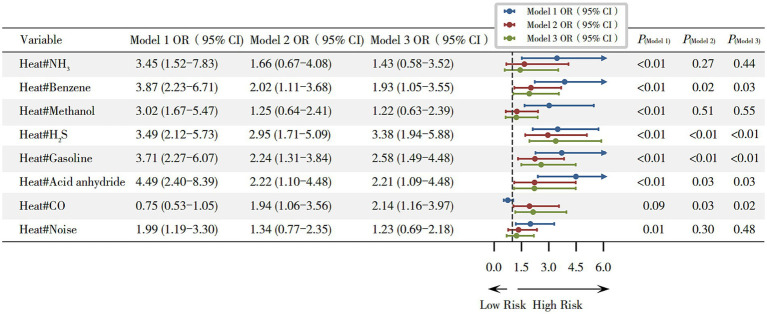
Joint effects of combined exposure to heat and other occupational hazards on hyperuricemia. Model 1 (unadjusted); Model 2 (adjusted for gender, age, and BMI); and Model 3 (adjusted for gender, age, and BMI, length of service, hyperglycemia, hypertension, smoking, and alcohol consumption). *p < 0.05.

In this study, we further examined the joint effects of heat and other occupational exposures on hyperuricemia. In the fully adjusted Model 3, results of multiplicative interaction analyses showed that heat combined with benzene (OR = 1.93, 95% CI 1.05–3.55), H_2_S (OR = 3.38, 95% CI 1.94–5.88), gasoline (OR = 2.58, 95% CI 1.49–4.48), acid anhydride (OR = 2.21, 95% CI 1.09–4.48) and CO (OR = 2.14, 95% CI 1.16–3.97) significantly increased the risk of hyperuricemia (all *p* < 0.05), as shown in Figure 3, while additive interaction analyses showed that these joint exposures did not exhibit significant positive additive effects (Supplementary Figure S3; Supplementary Table S3).

### 3.5 Development of hyperuricemia risk prediction model using machine learning algorithms

#### 3.5.1 Dataset split

To develop the hyperuricemia risk prediction model using machine learning algorithms, the dataset was split into a training set (*n* = 1,618) and a validation set (*n* = 694) in a 7:3 ratio. With the exception of noise exposure, no significant differences were observed in key demographic or baseline characteristics between the two datasets (*p* > 0.05), indicating good comparability. Detailed information is provided in Supplementary Table S4.

#### 3.5.2 Feature selection using LASSO regression

**Figure 4:**
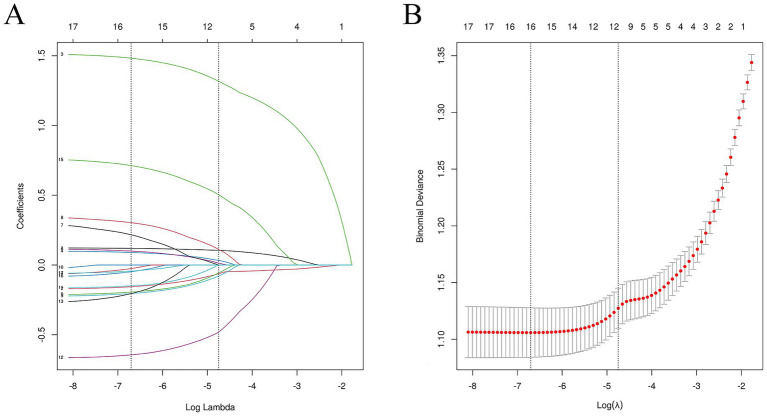
Feature selection of LASSO regression. **(A)** Shows the coefficient changes of LASSO regression; and **(B)** shows the fitting effect of LASSO regression.

Variables showing significant differences in the training set were entered into a LASSO regression to identify the most influential predictors of hyperuricemia. The optimal *λ* value corresponding to the minimum standard error distance was 0. 009, yielding 17 non-zero coefficient variables (Supplementary Table S6). The selected variables by LASSO regression include BMI, age, gender, smoking, alcohol drinking, length of service, benzene, noise, gasoline, and heat, used for model prediction and model construction. The results of the LASSO feature selection are shown in Figure 4.

#### 3.5.3 Model performance comparison

The results revealed that the Random Forest model achieved the highest AUC in the training set, while the LightGBM model performed best in the validation set. However, discrepancies between training and validation results suggested potential overfitting in the Random Forest model, whereas LightGBM exhibited superior generalizability and stability. Based on these findings, the LightGBM model was selected for subsequent SHAP-based interpretability analysis. Detailed results are provided in Supplementary Tables S7–S9 and Supplementary Figures S19,S20.

#### 3.5.4 LightGBM model training and evaluation

**Figure 5:**
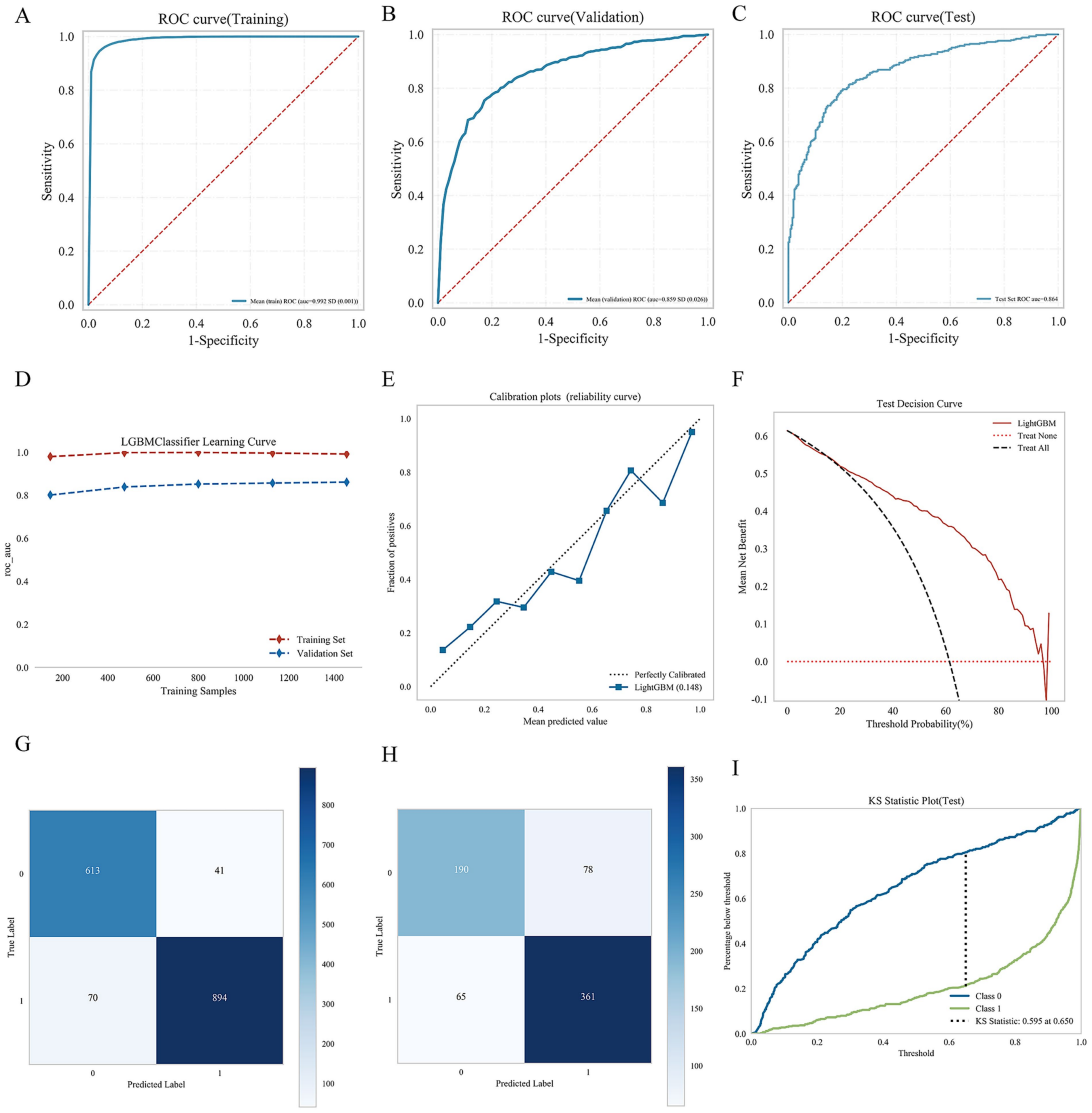
Consolidated performance evaluation of nine machine learning models. **(A)** Is ROC curve (Training set); **(B)** is ROC curve (Validation set); **(C)** is ROC curve (Test set); **(D)** is learning curve; **(E)** is calibration plots; **(F)** is test decision curve; **(G)** is confusion matrix of training set; **(H)** is confusion matrix of test set; and **(I)** is KS statistical diagram.

The ROC curve illustrates that LightGBM model achieved AUC value of 0.99 ± 0.01 for the training set, 0.86 ± 0.026 for the validation set, and 0.86 for the test set. The AUC difference between the validation and test sets was less than 10%, indicating good model generalization. The calibration curve showed good agreement between predicted probabilities and observed outcomes, with minor deviations at lower and midrange probability levels. Decision curve analysis (DCA) revealed that, across a wide range of threshold probabilities (0.1–0.7), the LightGBM model yielded a greater net benefit than both the “treat-all” and “treat-none” strategies. The confusion matrix indicated minimal risk of overfitting in the training set but a slightly elevated false-positive rate in the validation set. In addition, the KS statistics confirmed excellent discriminatory capacity and generalizability (KS = 0.60), with the optimal classification performance observed at a threshold of 0.65. Detailed results are shown in Figure 5.

#### 3.5.5 Quantifying the contribution of predictive features using SHAP values

**Figure 6:**
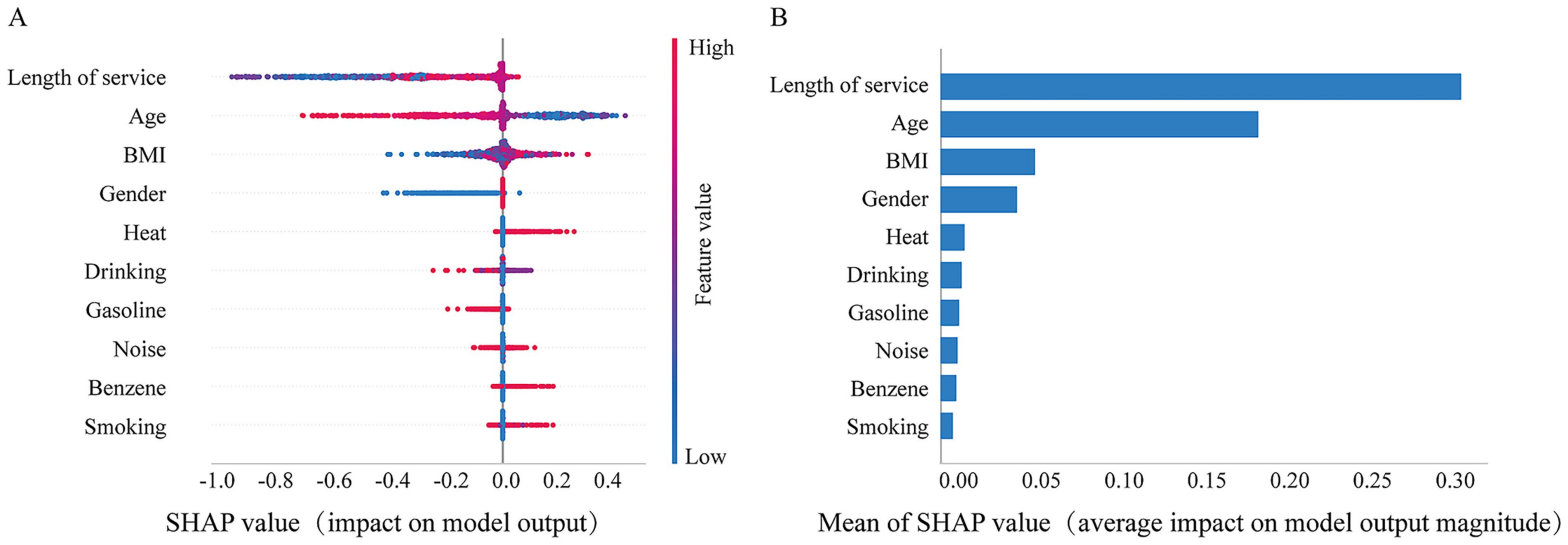
SHAP values quantify the contribution of predicted features. **(A)** The summary of SHAP values for variables in the LightGBM model. The horizontal axis represents the contribution of each feature to the model, while the vertical axis ranks the features based on the total sum of their SHAP values. The color indicates the feature value, with red corresponding to high values and blue corresponding to low values; and **(B)** is the histogram of feature importance for the LightGBM Model.

The results showed that the SHAP values for BMI and heat were positively associated with hyperuricemia, suggesting an increased risk of hyperuricemia with higher levels of these variables, whereas length of service and age showed a negative association. The bar chart represents the relative importance of each feature in the LightGBM model, with the top five most important features were length of service, age, BMI, gender, and heat exposure. Heat exposure was strongly occupational hazards associated with an increased risk of hyperuricemia. The detailed SHAP analysis results are shown in Figure 6.

## 4 Discussion

This study examined the association between occupational heat exposure and hyperuricemia among petrochemical workers. Our findings suggested that workplace heat exposure was associated with an increased risk of hyperuricemia. Using WQS regressions, we identified heat exposure as the most influential occupational hazard contributing to hyperuricemia risk. Additionally, a machine learning-based prediction model demonstrated high accuracy in forecasting hyperuricemia risk, with SHAP analysis confirmed the strong contribution of heat exposure.

Exposure to high temperatures has emerged as a significant public health and occupational health concern, particularly due to its association with renal impairment. Prolonged exposure to process-generated heat exacerbates thermal strain and predisposes workers to dehydration and kidney injury (35). Heat stress is a potential contributing factor to chronic kidney disease of unknown etiology (CKDu) in agricultural communities, which is increasingly recognized as a major cause of chronic kidney disease (36). This CKDu, also observed in Central America, often referred to as Mesoamerican nephropathy, has been epidemiologically and experimentally linked to recurrent dehydration and chronic heat exposure (37). Mounting evidence suggests that this condition may represent a distinct form of heat stress nephropathy (HSN), potentially exacerbated by global warming (38, 39). Furthermore, a myriad of chronic kidney disease cases has surfaced among agricultural workers and other individuals engaged in manual labor across various regions of the world (40). The disease is strongly correlated with the conditions of working and living in hot environments (41). Therefore, focusing on early indicators of renal impairment, such as hyperuricemia, may provide a valuable approach for early detection and prevention of heat-related kidney injury.

Petrochemical workers were routinely exposed to multiple occupational hazards such as heat, noise, and chemical toxins during the production and processing stages. Those occupational hazards may impact workers’ health (42). Salem et al. has reported elevated levels of urea, creatinine, and uric acid among Libyan petrochemical workers, suggesting subclinical or overt kidney dysfunction (43). Consistent with these findings, our results also revealed a high prevalence of hyperuricemia among petrochemical workers, which were significantly associated with heat exposure. Several factors may contribute to this high hyperuricemia prevalence. First, the study population consists of frontline occupational workers (male-dominated), who may inherently represent a higher-risk group and generally have higher uric acid levels. Second, the petrochemical industrial park is located in a coastal region, where dietary patterns often involve high consumption of seafoods (e.g., fish, shrimp, crab, and shellfish), leading to increased purine intake and elevated serum uric acid levels. Uric acid, the final product of purine metabolism in the body, is primarily excreted by the kidneys. Under normal physiological conditions, serum uric acid levels are maintained within a certain range. However, heat exposure may disrupt this balance through dehydration, reduced renal perfusion, and impaired excretory function (44), thereby increasing the risk of hyperuricemia. In workplaces that strictly follow occupational production norms and supervision of safety departments, the likelihood of petrochemical workers experiencing rapid and severe renal impairment is relatively low, while hyperuricemia is one of the early manifestations of renal dysfunction (45). Accordingly, attention to changes in uric acid levels and the onset of hyperuricemia may hold greater practical significance for safeguarding kidney health within this occupational population.

In this study, results of multistage GLM regression suggested a significant association between heat exposure and hyperuricemia, consistent with findings from salt pan workers, sugarcane field workers, and construction workers (46, 47). Despite differences in climate and work processes, heat exposure consistently elevates uric acid levels through biological mechanisms involving inflammation, oxidative stress, altered metabolism, and cytokine activation (48–50). Experimental evidence also supports this link, as animal studies have demonstrated that chronic heat exposure induces elevated serum uric acid (51). Interestingly, workers with hyperuricemia in our study tended to be younger and had shorter lengths of service. This unexpected finding may be attributed to a healthy worker survivor bias, whereby individuals unable to adapt to hazard exposures often leave high-exposure jobs earlier, and hiring restrictions might create a workforce with inherently higher health baseline levels (52, 53). Additionally, younger workers are frequently assigned to frontline positions, leading to higher exposure intensity despite shorter tenures (54), and longer-tenured workers may possess greater self-protective awareness due to extended training and job rotations, potentially reducing their risk of hyperuricemia.

In assessing joint effects of combined exposure to heat and other occupational hazards on hyperuricemia, significant multiplicative interaction effects were observed. Interestingly, the combined exposure to heat and gasoline or H_2_S, which both associated with higher hyperuricemia risk, despite their opposing individual effects. However, positive additive interactions were not observed, this finding consistent with Chen Y et al. (29), their research show that a multiplicative interaction rather than additive interaction between heat exposure and dust exposure in the development of hyperuricemia in steelworkers. A possible explanation of our study may lie in interaction mechanisms that modify physiological stress responses. While gasoline exposure alone may appear inversely associated with hyperuricemia, potentially due to residual confounding by work location, better ventilation, or health-based job selection, its co-occurrence with heat exposure may intensify oxidative and metabolic stress. Heat exposure promotes dehydration, reduced renal perfusion, and accumulation of reactive oxygen species (ROS) (55), which can impair uric acid excretion. Combined exposure to gasoline, containing volatile organic compounds (VOCs), may further exacerbate oxidative damage and inflammatory pathways, thereby amplifying renal tubular dysfunction (56, 57). This synergistic effect could result in a multiplicative increase in hyperuricemia risk, even when gasoline alone appears protective. Additionally, workers in high-temperature units often handle multiple chemical processes simultaneously, leading to greater overall exposure intensity and cumulative metabolic burden, which may also contribute to this combined effect. Krishnamurthy et al. found the potential synergistic effect between oil exposure and heat (58). The similar joint effect was observed for the combined exposure to heat and H₂S. This apparent paradox may reflect complex biological interactions and workplace exposure patterns. At low concentrations, H₂S can exert transient antioxidant effects by reduce the production of ROS (59). However, under conditions of heat stress, dehydration, and hypoxia, H₂S metabolism may shift toward pro-oxidative and cytotoxic pathways, enhancing oxidative stress and renal tubular injury (60). Furthermore, workers exposed to both heat and H₂S are typically engaged in frontline production units with higher overall exposure intensity and workload, which could amplify metabolic strain and renal burden (61). Therefore, combined exposure to heat and additional occupational hazards may further increase hyperuricemia risks among workers.

Traditional single-variable regression approaches often fall short in capturing the combined effects of multifactorial exposures. In contrast, the WQS model allows for estimation of cumulative effects while weighing each exposure’s relative contribution (62). Previous studies have used the WQS model to analyze the effects of mixed factors on hyperuricemia and to identify the most influential contributing factors (63). In this study, our WQS analysis identified heat and methanol exposure as the dominant contributors to hyperuricemia risk. The WQS model provides a better understanding of the relative impact of individual hazards on health outcomes and offers more precise strategies for pollutant control and management. Given the complexity of occupational exposures in petrochemical environments, predictive modeling plays an increasingly important role in occupational health surveillance. Machine learning models, particularly those that capture non-linear relationships and interactions, offer substantial advantages over traditional statistical approaches (64–66). Anttila et al. has already explored the risk prediction modeling in petrochemical workers, with findings indicating that exposure to hydrocarbons in crude oil may increase the risk of kidney cancer by up to threefold (67). In this study, we comparison nine supervised learning models to predict hyperuricemia risk. After cross-validation and performance evaluation, the LightGBM model was selected for its superior balance of accuracy and generalizability.

The LightGBM model demonstrated strong predictive performance, but the AUC results suggest potential overfitting. In practical terms, this predictive model may be incorporated into occupational health surveillance systems to support dynamic risk stratification and provide early indications of workers at higher risk of hyperuricemia. Such integration could help inform timely preventive measures. Nevertheless, further external validation and real-world implementation studies are warranted before real-world application. While machine learning algorithms offer strong predictive performance, they are often criticized for their lack of transparency (68). To address this, we incorporated SHAP analysis, which has been widely adopted for interpreting machine learning models. By calculating the marginal contribution of each feature to the prediction outcome, SHAP effectively quantified the impact of individual variables (69). SHAP results confirmed that BMI and heat exposure are dominant risk factors, offering actionable insights for workplace surveillance and intervention prioritization.

Although this study identified an association between occupational heat exposure and hyperuricemia among petrochemical workers, several limitations should be acknowledged. First, this research was derived from retrospective health examination records, and we adopted a complete-case analysis approach for all main exposure and outcome variables, which may reduce sample size. The inability to infer causality is also inherently limited. Second, our study did not capture information on dietary habits, hydration patterns, genetic predisposition, medication use, metabolic factors, use of personal protective equipment, medication use, and acclimatization, which may influence the assessment of heat-hyperuricemia association. Third, heat exposure in this study primarily refers to process-generated heat from petrochemical production. Workers’ occupational health examinations were conducted during the warmer months (March to August) each year, which may potentially overestimate the association between heat exposure and hyperuricemia. Nevertheless, the uniform health examination period across all workers helps to minimize between-individual seasonal variability and thus strengthens internal comparability. However, it should be noted that the lack of year-round health examination data may limit our ability to fully characterize seasonal variations in the heat–hyperuricemia relationship. Fourth, during the additive interaction analysis in this study, some Synergy Index (SI) values appeared as NaN, likely because the odds ratio (OR) for a single exposure equaled 1, resulting in a zero denominator in the SI formula. To obtain valid estimates, we followed the software recommendation and set recode = TRUE to reverse-code the exposures. This adjustment ensured computational validity but may have altered the directionality of the additive interaction estimates (negative). Therefore, the opposite directions observed between additive and multiplicative interactions in this study should be interpreted with caution, as they may partly reflect a computational artifact rather than a genuine antagonistic interaction on the additive scale. Finally, the absence of creatinine/eGFR, albuminuria, temperature monitoring during the production process as well as relevant information regarding the workers’ production departments, has precluded a more refined assessment of the association between heat exposure (in terms of intensity and duration) and additional biomarkers of early renal dysfunction.

To address these limitations, our ongoing prospective cohort study has been designed to incorporate structured occupational and dietary questionnaires to systematically collect data on exposure histories and lifestyle factors, as well as surveying nearby residents as a control group to better evaluate the health effects of occupational exposures. Furthermore, we are also actively collaborating with government monitoring agencies to obtain accurate measurements of exposure levels and duration for various occupational hazards, and model dose–response relationships using methods such as restricted cubic splines. These efforts aim to enhance the robustness of future analyses and to inform evidence-based strategies for protecting the health of petrochemical workers.

## 5 Conclusion

Occupational heat exposure is a prevalent hazard in the petrochemical industry and is significantly associated with hyperuricemia. By integrating epidemiological and machine learning methods, this study quantified both the independent and combined effects of heat and other hazards on kidney health and identified key predictors of hyperuricemia risk. Findings of this study highlight the need for targeted preventive measures, including heat stress reduction and proactive monitoring high-risk workers. Future research should explore the biological mechanisms underlying these associations and externally validate predictive models in diverse occupational settings to better prevent heat-related early renal dysfunction.

## Data Availability

The original contributions presented in the study are included in the article/Supplementary material, further inquiries can be directed to the corresponding authors.
