# Supplementary material for: Association between occupational heat exposure and early renal dysfunction among Chinese petrochemical workers: a combined machine learning and WQS modeling study
Source: Front Public Health. 2025 Nov 12;13:1648619. doi: 10.3389/fpubh.2025.1648619 (PMC12647014; doi:10.3389/fpubh.2025.1648619)
Supplement: Supplementary file 1 [file Data_Sheet_1.docx]

**Supplementary Material**

**1. Supplementary analysis of the association between heat exposure and hyperuricemia**

## 1.1 Causal Directed Acyclic Graph (DAG)

To justify the adjustment sets, we constructed a causal DAG based on prior literature and the variables included in our analysis, illustrating the relationship between heat exposure and hyperuricemia, for better identification of potential bias pathways and confounders (e.g., BMI, smoking), thereby enabling more accurate causal inference, the details as shown in Figure S1.


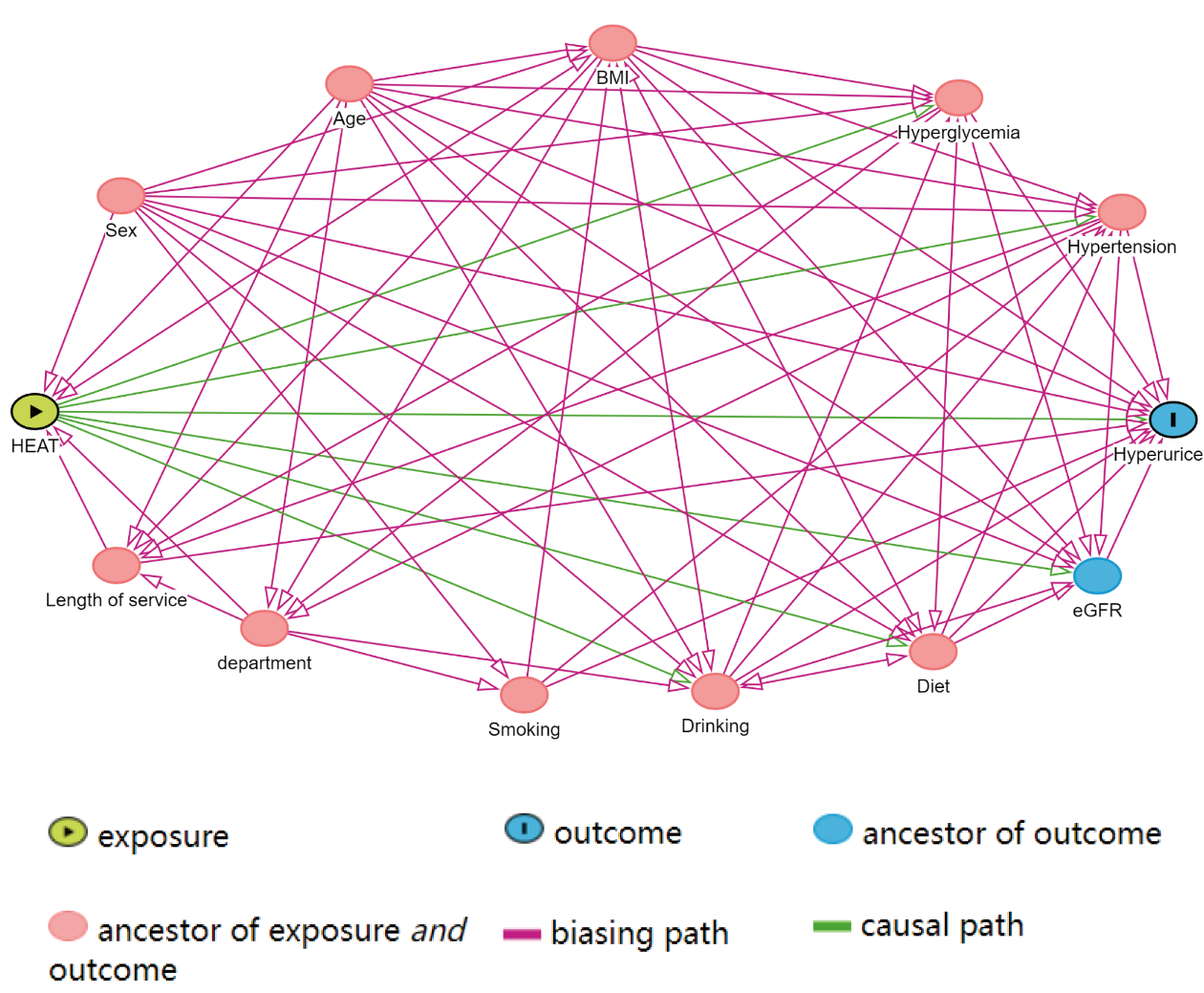


Figure S1 Causal directed acyclic graph depicting the relationship between heat exposure and hyperuricemia

## 1.2 E-value calculations for the primary heat–hyperuricemia association.

## To assess the potential influence of unmeasured confounders, we conducted E-value analyses for the association between heat exposure and hyperuricemia. The E-values for OR were 4.91 in Model 1, 2.53 in Model 2, and 2.74 in Model 3. These results suggest that, in the Model 3, an unmeasured confounder would need to be associated with both occupational heat exposure and hyperuricemia by a risk ratio of at least 2.74 each, beyond the measured covariates, to fully explain away the observed association. The details are shown in Table S1.

Table S1 E-value calculations of heat–hyperuricemia association.

| Model | OR (95% CI) | E-value for OR | E-value for CI |
| --- | --- | --- | --- |
| Model 1 | 2.73（2.17‒3.45） | 4.91 | 3.75 |
| Model 2 | 1.58（1.21‒2.06） | 2.53 | 1.71 |
| Model 3 | 1.68（1.28‒2.20） | 2.74 | 1.87 |

Note: Model 1 (unadjusted model); Model 2 (basic adjusted model) was adjusted for gender, age, and BMI; Model 3 (fully adjusted) was further adjusted for additional confounders, including length of service, hyperglycemia, hypertension, smoking, and alcohol consumption.

1.3 Adjust heat as a covariate into the models of chemical exposures to examine the association between occupational hazards and hyperuricemia

To examine whether the negative coefficients persisted, we incorporated heat as a covariate into the analyses of chemical exposures, while adjusting for the same covariates as in the models. In the fully adjusted model, exposures to H₂S (OR = 0.61, 95% CI: 0.49–0.77), gasoline (OR = 0.52, 95% CI: 0.41–0.65), and CO (OR = 0.70, 95% CI: 0.51–0.95) were associated with lower odds of hyperuricemia, the details as shown in Figure S2.


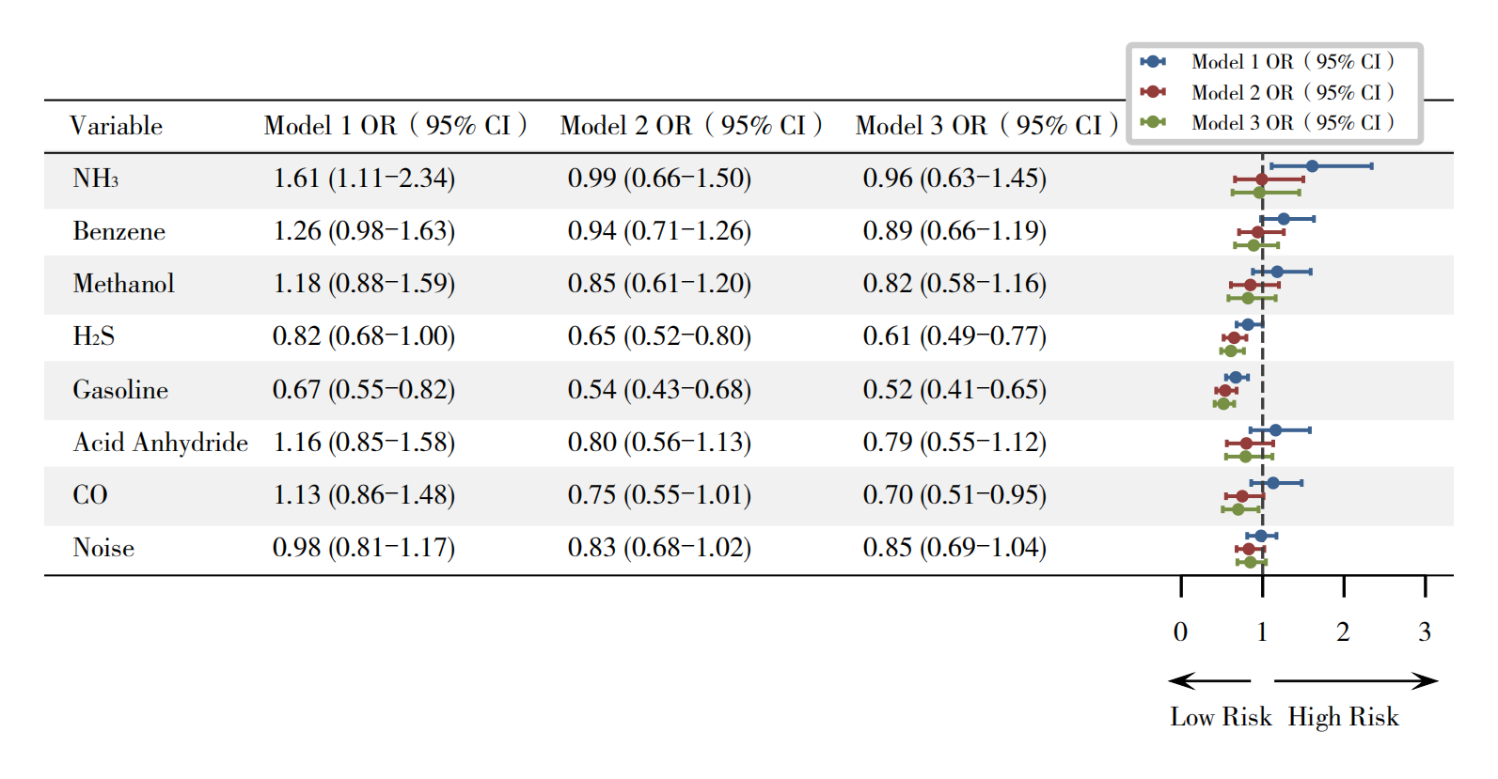


Figure S2 Adjust heat as a covariate into the model examining the association between occupational hazards and hyperuricemia

Note: Model 1 (adjusted for heat); Model 2 (adjusted for heat, gender, age, and BMI); Model 3 (further adjusted for additional confounders, including length of service, hyperglycemia, hypertension, smoking, and alcohol consumption).

1.4 The counts of each exposure factor in combined exposure

In the combined exposure analysis, heat exposure frequently co-occurred with multiple occupational hazards. Among participants with hyperuricemia, those with heat exposure showed markedly higher counts of co-exposures compared with those without heat exposure. In contrast, control group generally exhibited lower co-exposure counts, suggesting that petrochemical workers are frequently exposed to multiple occupational hazards concurrently during production. The details are shown in Table S2.

Table S2 The counts of each exposure factor in combined exposure [n (%)]

|  | Control (n=922) | | Hyperuricemia (n=1390) | |
| --- | --- | --- | --- | --- |
| Variable | Heat exposure | | Heat exposure | |
|  | NO (n=814) | YES (n=108) | NO (n=1020) | YES (n=370) |
| NH_3_ |  |  |  |  |
| No | 796 (97.79) | 66 (61.11) | 1,005 (98.53) | 151 (40.81) |
| Yes | 18 (2.21) | 42 (38.89) | 15 (1.47) | 219 (59.19) |
| Benzene |  |  |  |  |
| No | 727 (89.31) | 73 (67.59) | 931 (91.27) | 149 (40.27) |
| Yes | 87 (10.69) | 35 (32.41) | 89 (8.73) | 221 (59.73) |
| Methanol |  |  |  |  |
| No | 760 (93.37) | 67 (62.04) | 971 (95.20) | 160 (43.24) |
| Yes | 54 (6.63) | 41 (37.96) | 49 (4.80) | 210 (56.76) |
| H_2_S |  |  |  |  |
| No | 579 (71.13) | 46 (42.59) | 804 (78.82) | 90 (24.32) |
| Yes | 235 (28.87) | 62 (57.41) | 216 (21.18) | 280 (75.68) |
| Gasoline |  |  |  |  |
| No | 592 (72.73) | 51 (47.22) | 855 (83.82) | 118 (31.89) |
| Yes | 222 (27.27) | 57 (52.78) | 165 (16.18) | 252 (68.11) |
| Acid anhydrides |  |  |  |  |
| No | 764 (93.86) | 68 (62.96) | 985 (96.57) | 152 (41.08) |
| Yes | 50 (6.14) | 667 (37.04) | 35 (3.43) | 218 (58.92) |
| CO |  |  |  |  |
| No | 739 (90.79) | 58 (53.70) | 948 (92.94) | 127 (34.32) |
| Yes | 75 (9.21) | 667 (46.30) | 72 (7.06) | 243 (65.68) |
| Noise |  |  |  |  |
| No | 535 (65.72) | 36 (33.33) | 698 (68.43) | 82 (22.16) |
| Yes | 279 (34.28) | 72 (66.67) | 322 (31.57) | 288 (77.84) |

1.5 Additive interaction effects of heat exposure and other occupational hazards

Most additive interaction indicators (RERI, AP, SI) for heat exposure combined with other occupational hazards were negative or below 1, suggesting that these joint exposures did not exhibit positive additive effects (Note: In the original manuscript, some SI values appeared as NaN, likely because the OR for a single exposure equaled 1, resulting in a zero denominator in the SI formula. For the re-analysis, to obtain valid estimates, we followed the system recommendation and set recode = TRUE to reverse-code the exposures, thereby ensuring that the estimates reflected the true direction of interaction). The details are shown in Table S3 and Figure S3.

Table S3 Additive interaction effects of combined exposure to heat and other occupational hazards on hyperuricemia

| Variable | *RERI* (95%CI) | *AP* (95%CI) | *SI* (95%CI) |
| --- | --- | --- | --- |
| Heat#NH_3_ | -0.45 (-3.84 ‒ 0.94) | -0.20 (-1.00 ‒ 0.44) | 0.73 (0.32 ‒ 1.69) |
| Heat#Benzene | -1.13 (-3.02 ‒ 0.02) | -0.59 (-1.61 ‒ -0.01) | 0.45 (0.19 ‒ 1.03) |
| Heat#Methanol | -0.13 (-1.81 ‒ 1.01) | -0.06 (-0.74 ‒ 0.37) | 0.91 (0.39 ‒ 2.13) |
| Heat#H_2_S | -2.34 (-4.07 ‒ -0.96) | -1.18 (-2.54 ‒ -0.45) | 0.30 (0.13 ‒ 0.69) |
| Heat#Gasoline | -1.63 (-3.51 ‒ 0.00) | -0.55 (-1.44 ‒ -0.04) | 0.55 (0.30 ‒ 1.02) |
| Heat#Acid anhydride | -1.33 (-4.42 ‒ 0.24) | -0.45 (-1.31 ‒ 0.06) | 0.59 (0.32 ‒ 1.10) |
| Heat#CO | -1.24 (-3.45 ‒ 0.28) | -0.44 (-1.30 ‒ 0.05) | 0.59 (0.31 ‒ 1.13) |
| Heat#Noise | -0.23 (-1.25 ‒ 0.91) | -0.12 (-0.97 ‒ 0.26) | 0.79 (0.27 ‒ 2.33) |


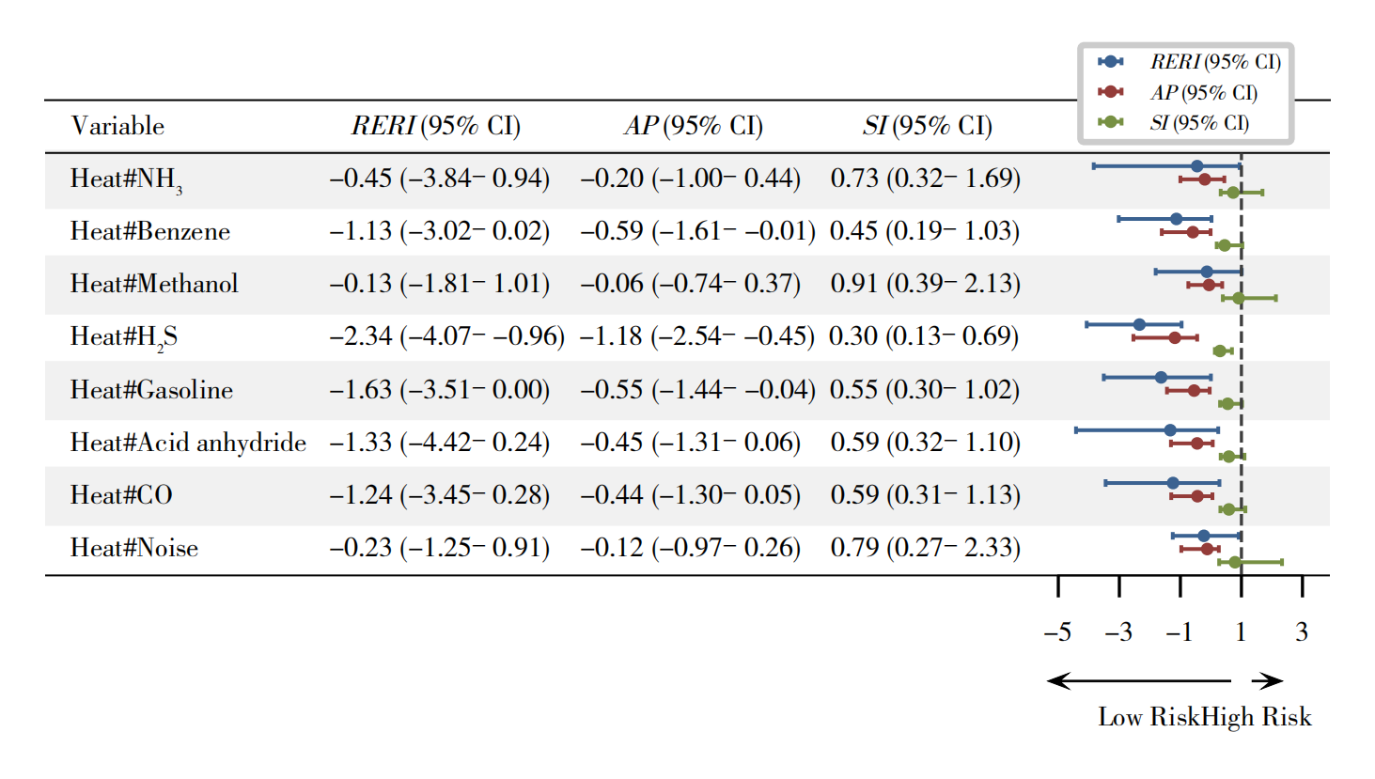


Figure S3 Additive interaction effects of heat exposure and other occupational hazards

1.6 Sensitivity analysis

To further investigate the association between occupational hazard exposure and hyperuricemia among petrochemical workers, we performed stratified analyses by sex, age, BMI and length of service.

(1) Sex-stratified analysis

Sex-stratified analyses showed that the prevalence of hyperuricemia was approximately 33.4% in females and 70.9% in males, both markedly higher than the 20–30% typically reported in the general population. Several factors may contribute to this elevated prevalence. First, the study population primarily consists of frontline occupational workers (male-dominated), who may inherently represent a higher-risk group due to greater exposure intensity and work-related stressors. Second, the petrochemical industrial park is located in a coastal region, where local dietary patterns often involve high consumption of seafood (e.g., fish, shrimp, crab, shellfish), leading to increased purine intake and elevated serum uric acid levels.

In the female-specific analyses, heat exposure was significantly associated with hyperuricemia in the unadjusted Model 1 (OR = 2.31, 95% CI: 1.48–3.61). This association was no longer statistically significant after adjusted Model 2 (OR = 1.53, 95% CI: 0.94–2.49), and Model 3 (OR = 1.58, 95% CI: 0.96–2.59). Similarly, positive associations were observed for NH₃, benzene, methanol, and CO in unadjusted models, but these associations became non-significant after adjustment. Detailed results are shown in Figure S4.

In the male-specific analyses, heat exposure showed a consistent positive association with hyperuricemia across all models, with the Model 3 yielding an OR of 1.65 (95% CI: 1.18–2.28). Additionally, exposure to H₂S (OR = 0.69, 95% CI: 0.54–0.88) and gasoline (OR = 0.50, 95% CI: 0.38–0.65) was associated with lower odds of hyperuricemia. More detailed results are shown in Figure S5.

In summary, the results indicate that heat exposure was significantly and positively associated with hyperuricemia in the unadjusted model. These findings are consistent with the results presented in the main text of this study, with detailed results shown in Figure S4 and S5.


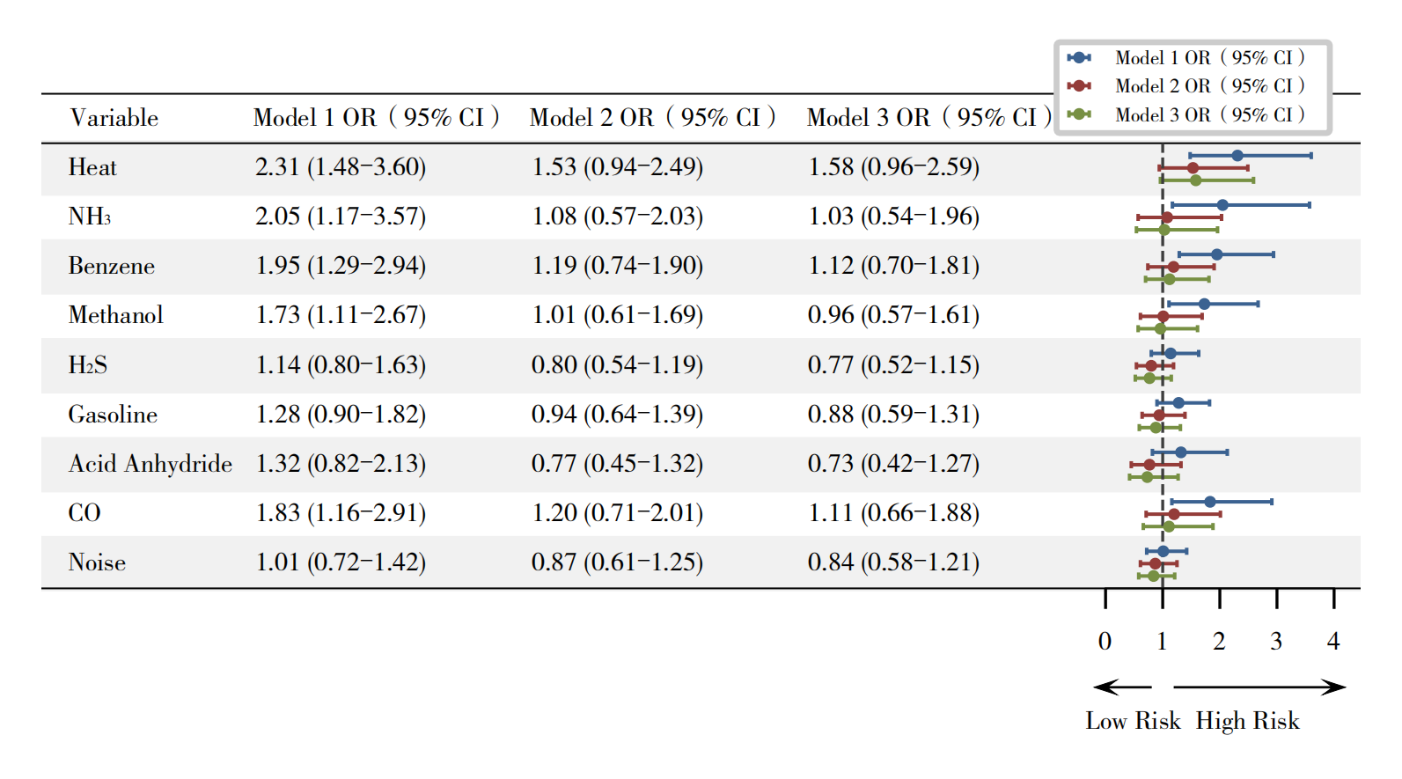


Figure S4 The association between occupational hazard exposures and hyperuricemia among female workers.


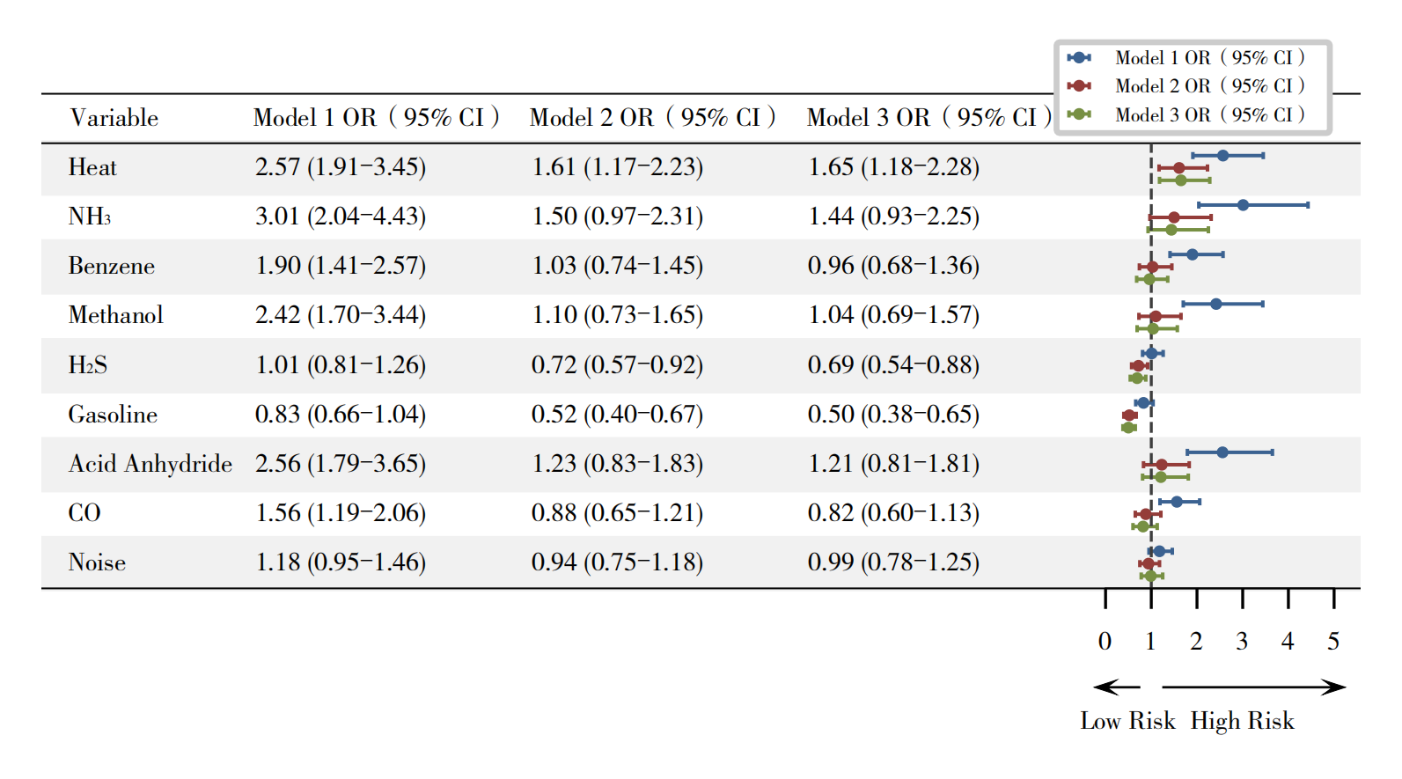


Figure S5 The association between occupational hazard exposures and hyperuricemia among male workers.

(2) Age-stratified analysis

The study population was divided into three subgroups (< 30, 30-45, and >45 years) based on age quantiles. In the <30 subgroup, all occupational exposures were positively associated with hyperuricemia in the unadjusted model, and most associations remained significant after full adjustment, except for H_2_S and gasoline. Among workers aged 30–45 years, positive associations were observed for heat (OR = 3.08, 95% CI: 1.30–7.30) and H_2_S (OR = 2.15, 95% CI: 1.09–4.24) after adjustment, while methanol showed positive but highly uncertain estimates due to wide confidence intervals, likely reflecting limited sample size. In the >45 subgroup, heat exposure was positively associated with hyperuricemia in the unadjusted Model 1 (OR = 1.86, 95% CI: 1.22–2.83) and Model 2 (OR = 1.76, 95% CI: 1.13–2.73), whereas gasoline (OR = 0.60, 95% CI: 0.44–0.84) and noise (OR = 0.74, 95% CI: 0.55–0.99) were inversely associated after adjustment. After age-standardization using the China 2010 standard population, the overall prevalence of hyperuricemia was 53.7%. In summary, heat exposure showed consistent positive associations with hyperuricemia across all age strata, while inverse associations for gasoline and hydrogen sulfide were evident in specific subgroups, consistent with the univariate findings. The detailed results are shown in Figures S6-8.


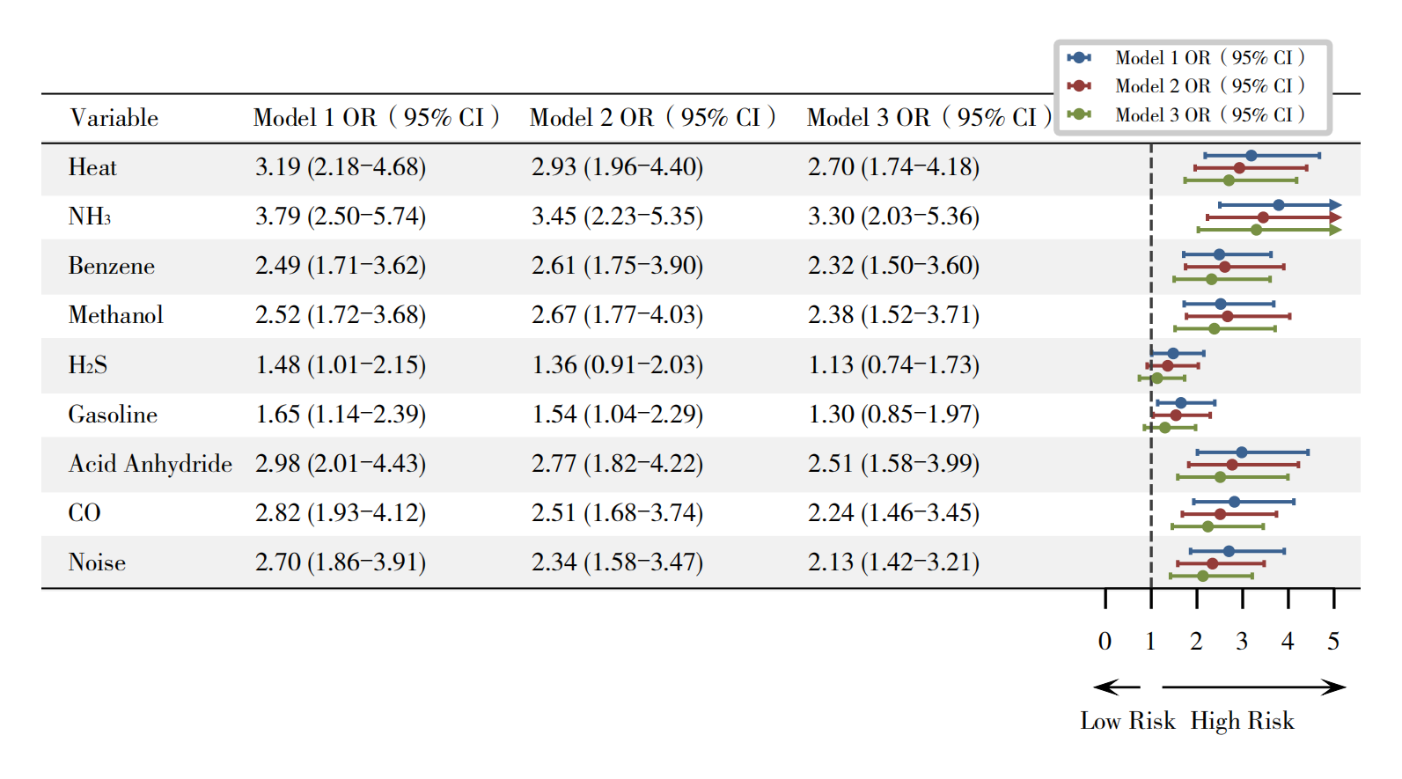


Figure S6 Age-stratified analysis (<30 years) of the association between occupational hazard exposures and hyperuricemia.


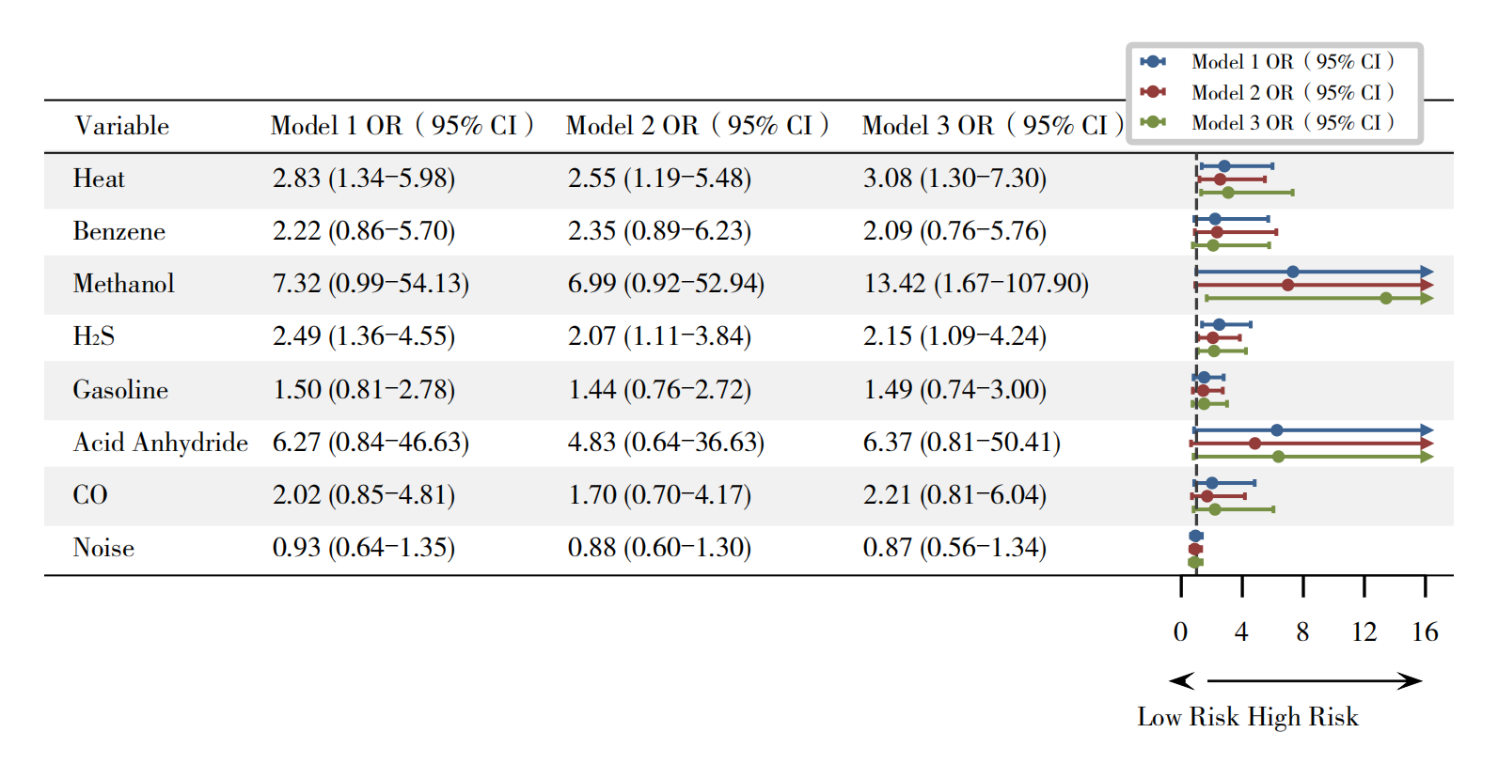


Figure S7 Age-stratified analysis (30-45 years) of the association between occupational hazard exposures and hyperuricemia.

Note: Due to limited sample size in this age group, reliable estimation of the upper confidence interval limit was not possible. Therefore, the results for NH₃ were omitted from the forest plot.


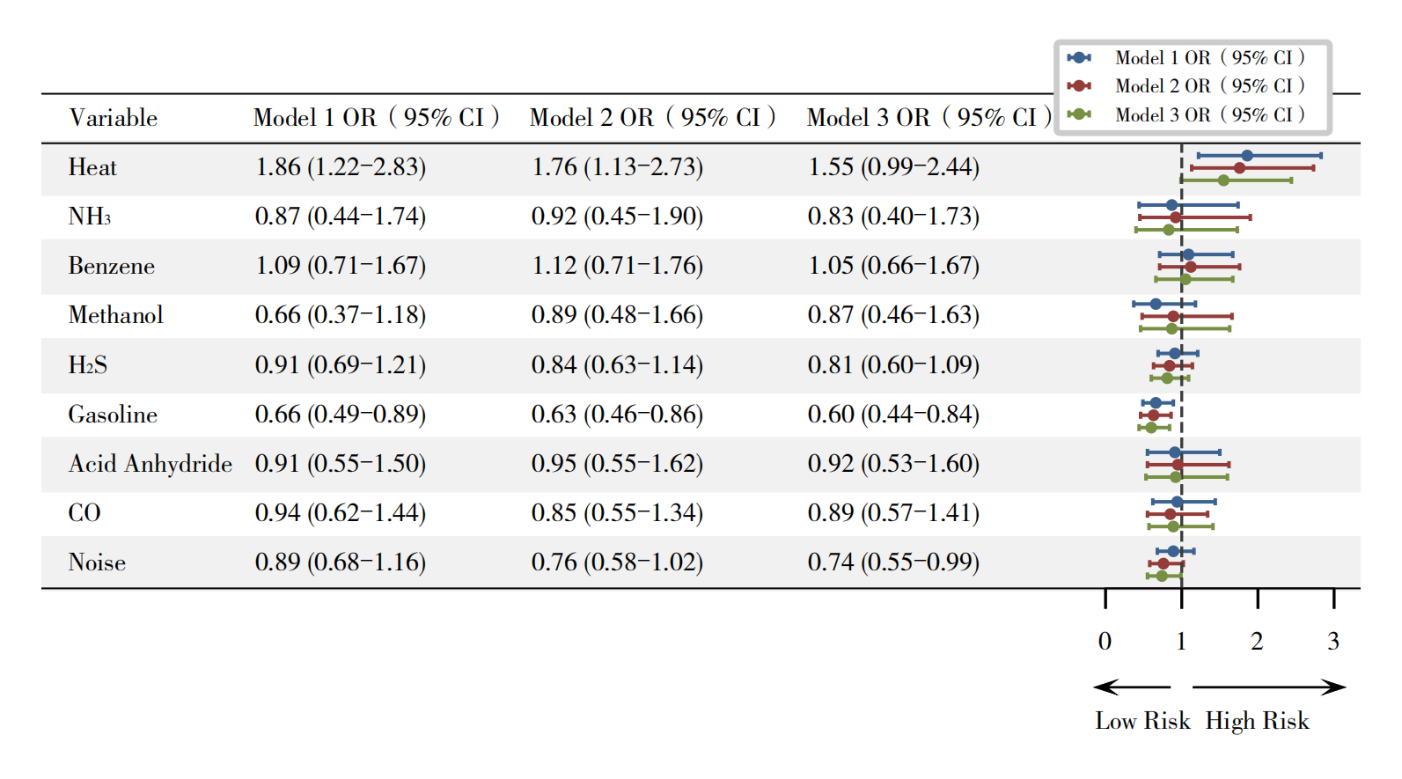


Figure S8 Age-stratified analysis (>45 years) of the association between occupational hazard exposures and hyperuricemia.

(3) BMI-stratified analysis

According to cut-off points for Chinese adults, the study population was categorized into three BMI subgroups: < 24, 24–28 (overweight), and > 28 (obese). In the BMI < 24 subgroup, all occupational exposures except H_2_S and gasoline were positively associated with hyperuricemia in the unadjusted model, and heat remained significant after full adjustment (OR = 1.80, 95% CI: 1.28–2.54). In the BMI 24–28 subgroup, heat showed positive association in the unadjusted model (OR = 2.12, 95% CI: 1.37–3.28). In the BMI >28 subgroup, heat remained positive association in the unadjusted Model 1 (OR = 2.77, 95% CI: 1.20–6.35), while NH_3_ and CO also indicated a positive trend; however, its wide confidence interval suggested substantial uncertainty, likely due to the limited sample size. The detailed results are shown in Figures S9-11.


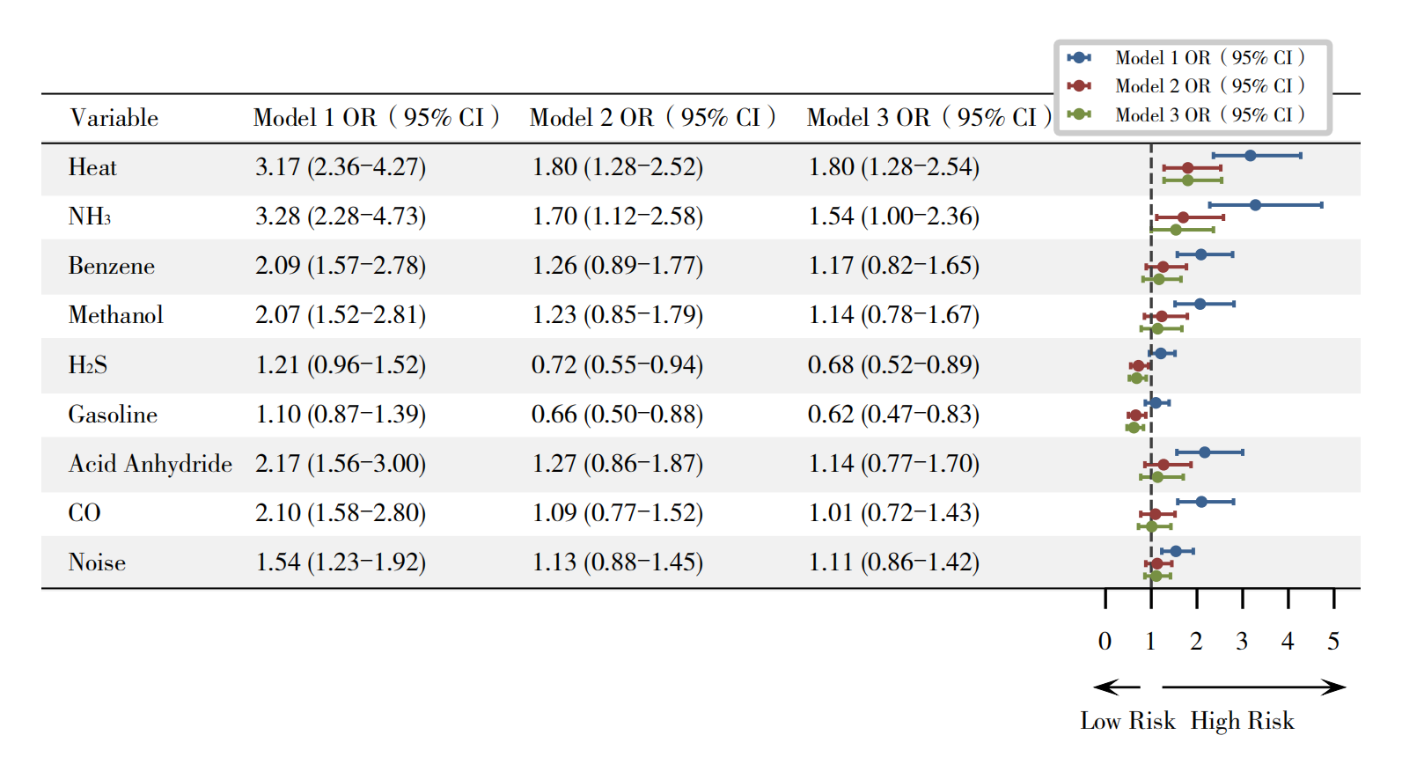


Figure S9 BMI-stratified analysis (BMI < 24) of the association between occupational hazard exposures and hyperuricemia.


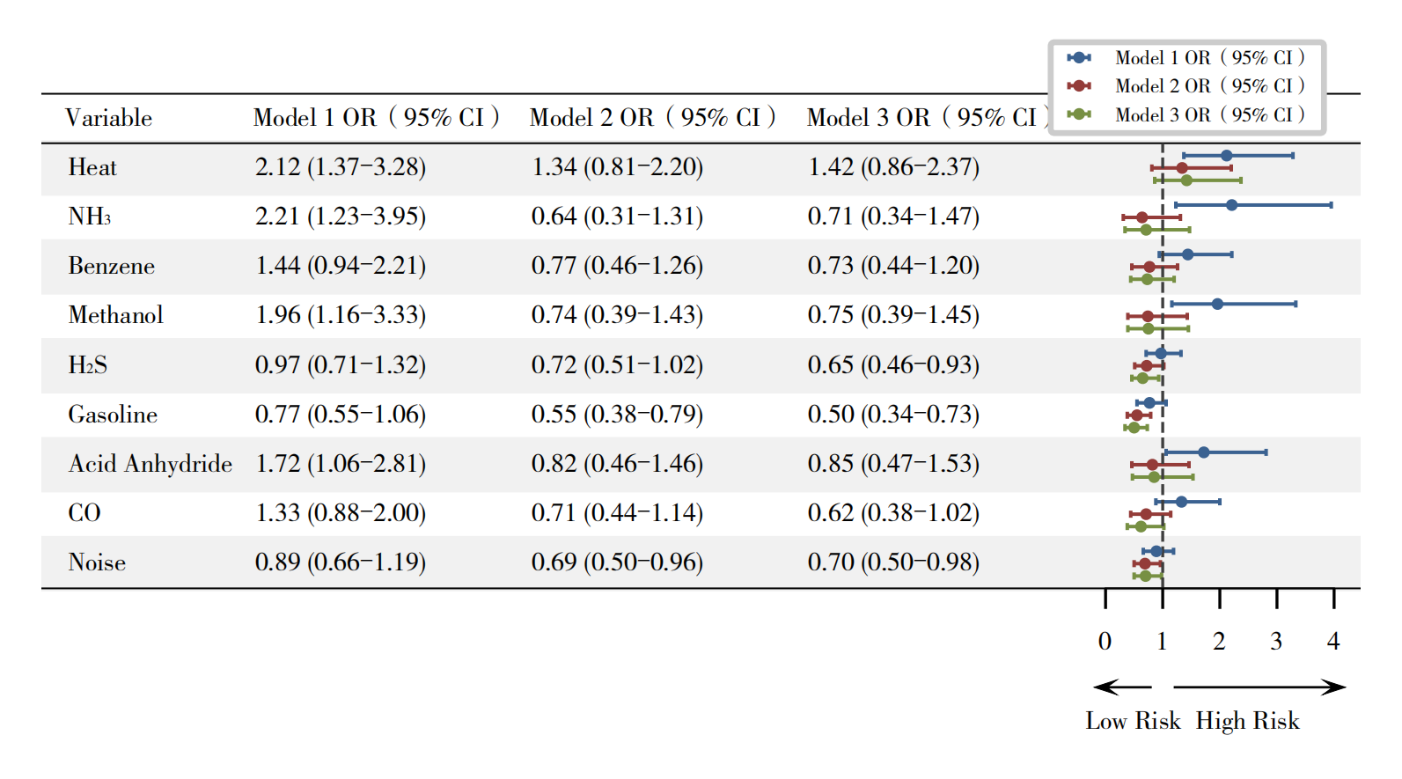


Figure S10 BMI-stratified analysis (24*-*28) of the association between occupational hazard exposures and hyperuricemia.


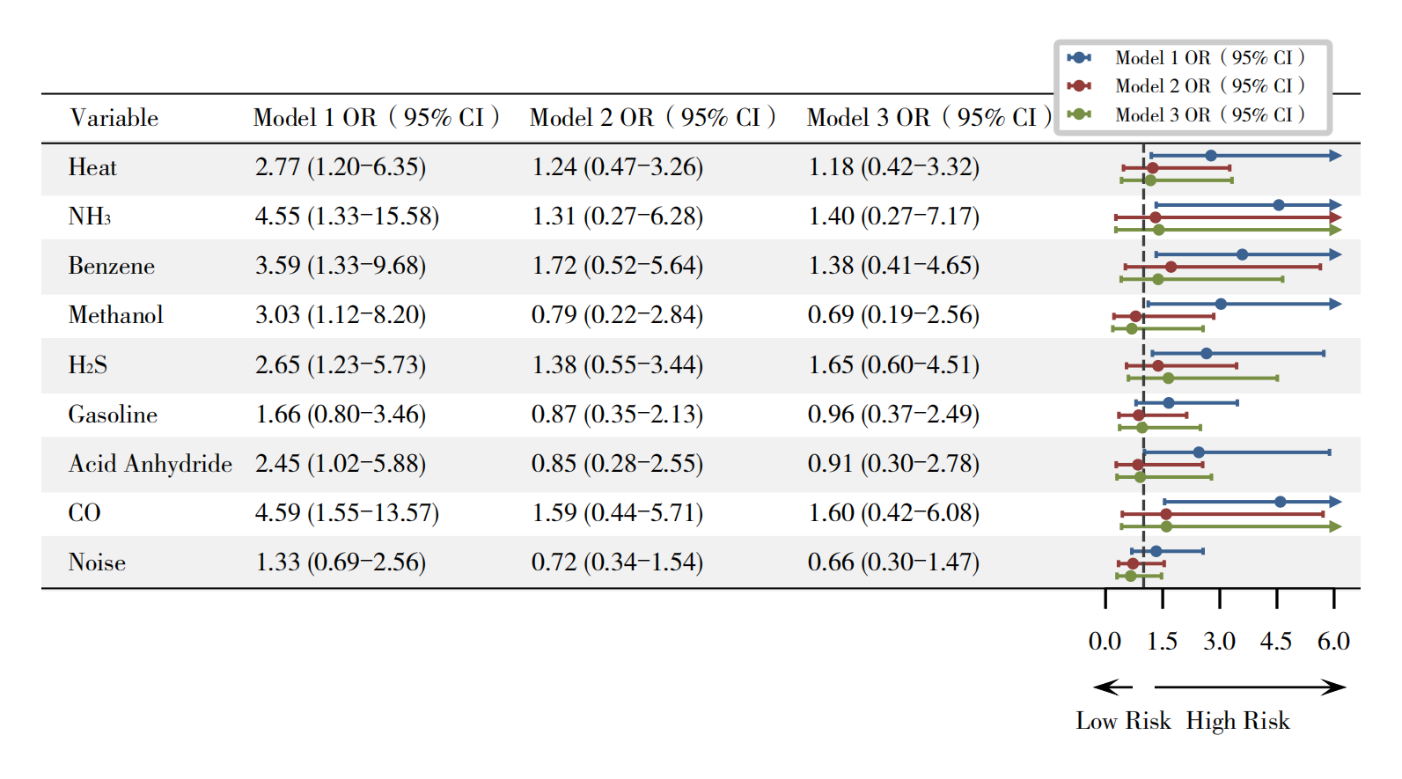


Figure S11 BMI-stratified analysis (> 28) of the association between occupational hazard exposures and hyperuricemia.

(4) Length of service-stratified analysis

The study population was divided into two subgroups (≤ 10 and >10 years) based on length of service. In the length of service ≤ 10 subgroup, all occupational exposures were positively associated with hyperuricemia in the unadjusted model, and most associations remained significant after full adjustment except for H_2_S and gasoline.

I In the length of service >10 years subgroup, positive associations were observed for heat (OR = 2.12, 95% CI: 1.50–2.99) in the unadjusted model, and positive associations remained significant in the adjusted Model 2: heat (OR =1.87, 95% CI: 1.26–2.76), and Model 3: heat (OR = 1.93, 95% CI: 1.30–2.88). These findings are consistent with our previous analysis of the association between occupational hazards and hyperuricemia, suggesting a significant association between heat exposure and hyperuricemia. The detailed results are shown in Figures S12-13.


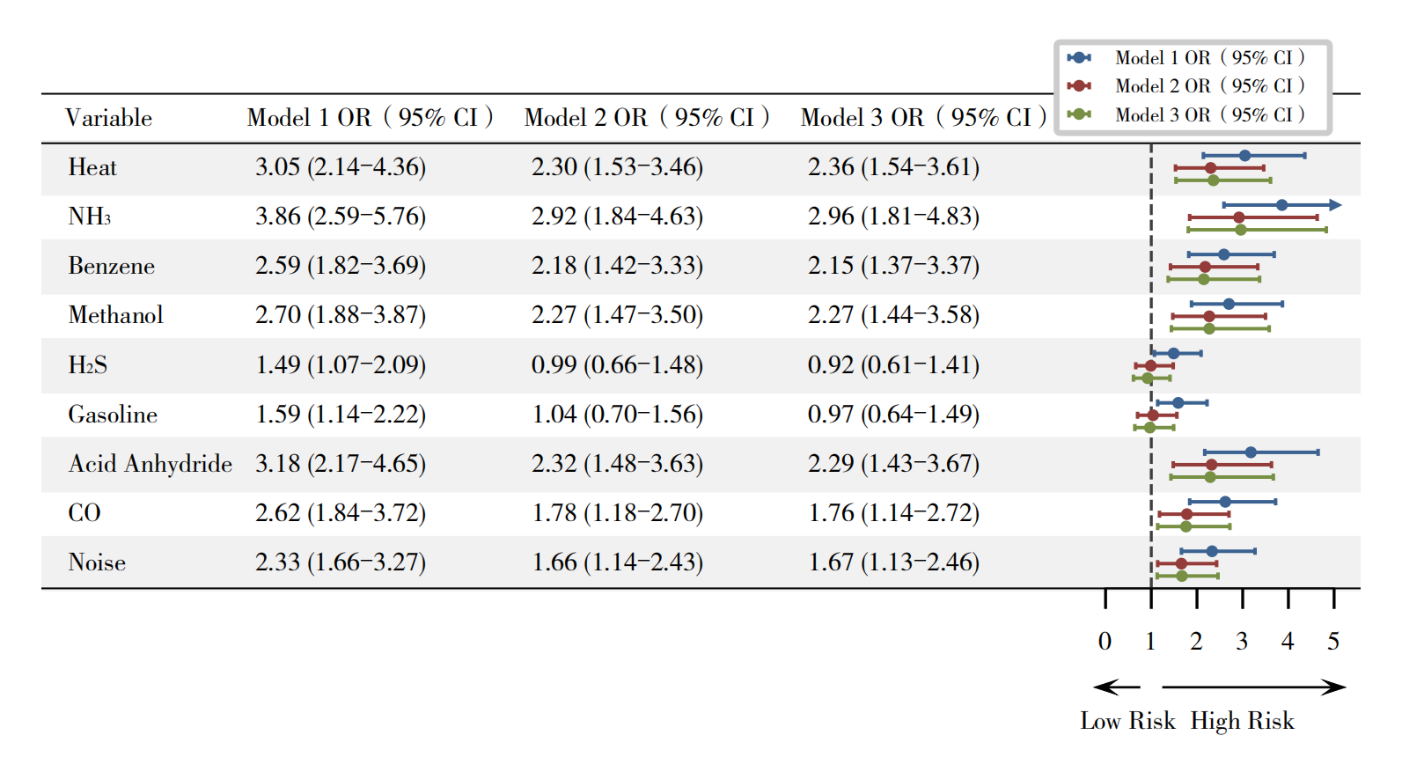


Figure S12 Length of service -stratified analysis (≤ 10 years) of the association between occupational hazard exposures and hyperuricemia.


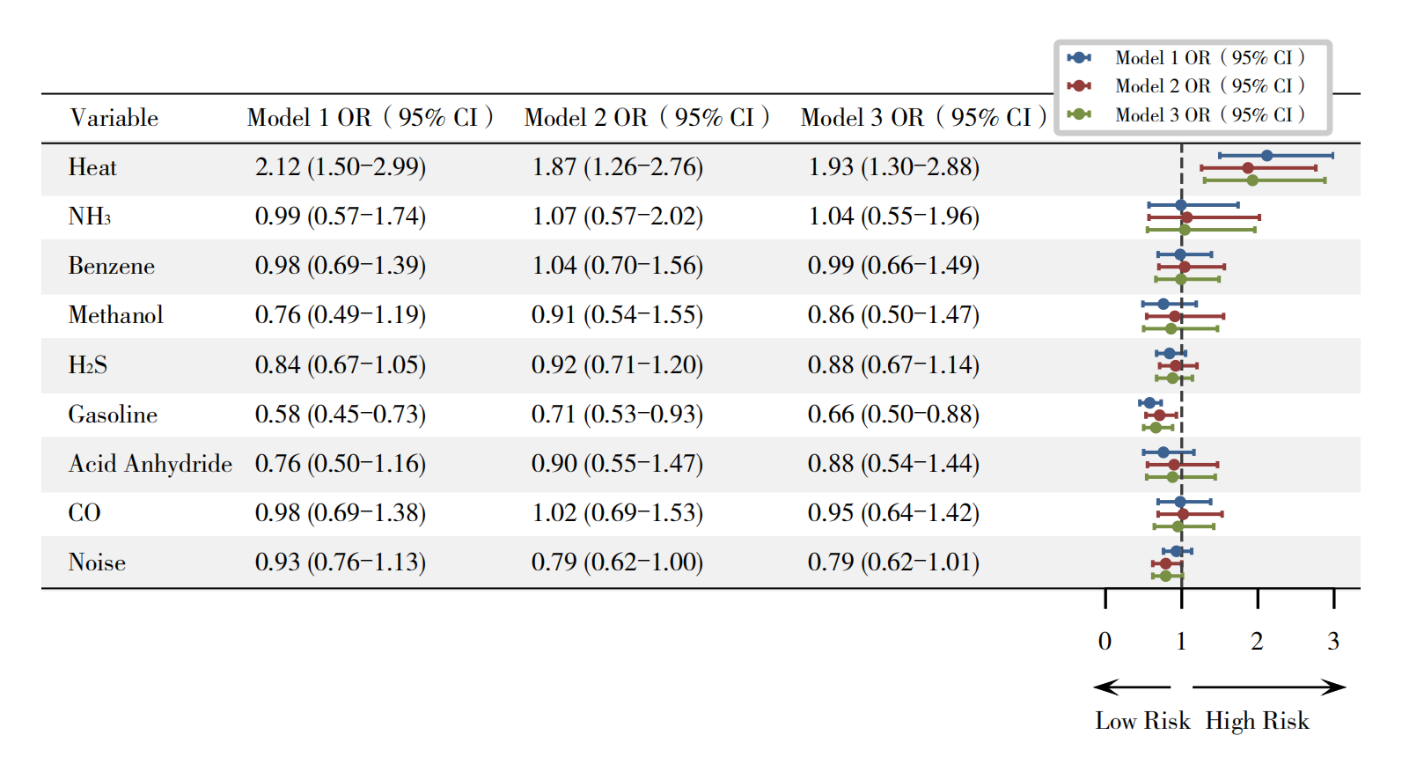


Figure S13 Length of service-stratified analysis (>10 years) of the association between occupational hazard exposures and hyperuricemia.

**2. WQS model analysis**

2.1 Negative-direction analysis of WQS model

When the WQS model was re-specified without constraining the direction of association, gasoline, noise, and hydrogen sulfide accounted for the largest negative weights (84.6%, 6.2%, and 4.4%, respectively), suggesting potential inverse relationships with hyperuricemia. The detailed results are shown in Figure S14.


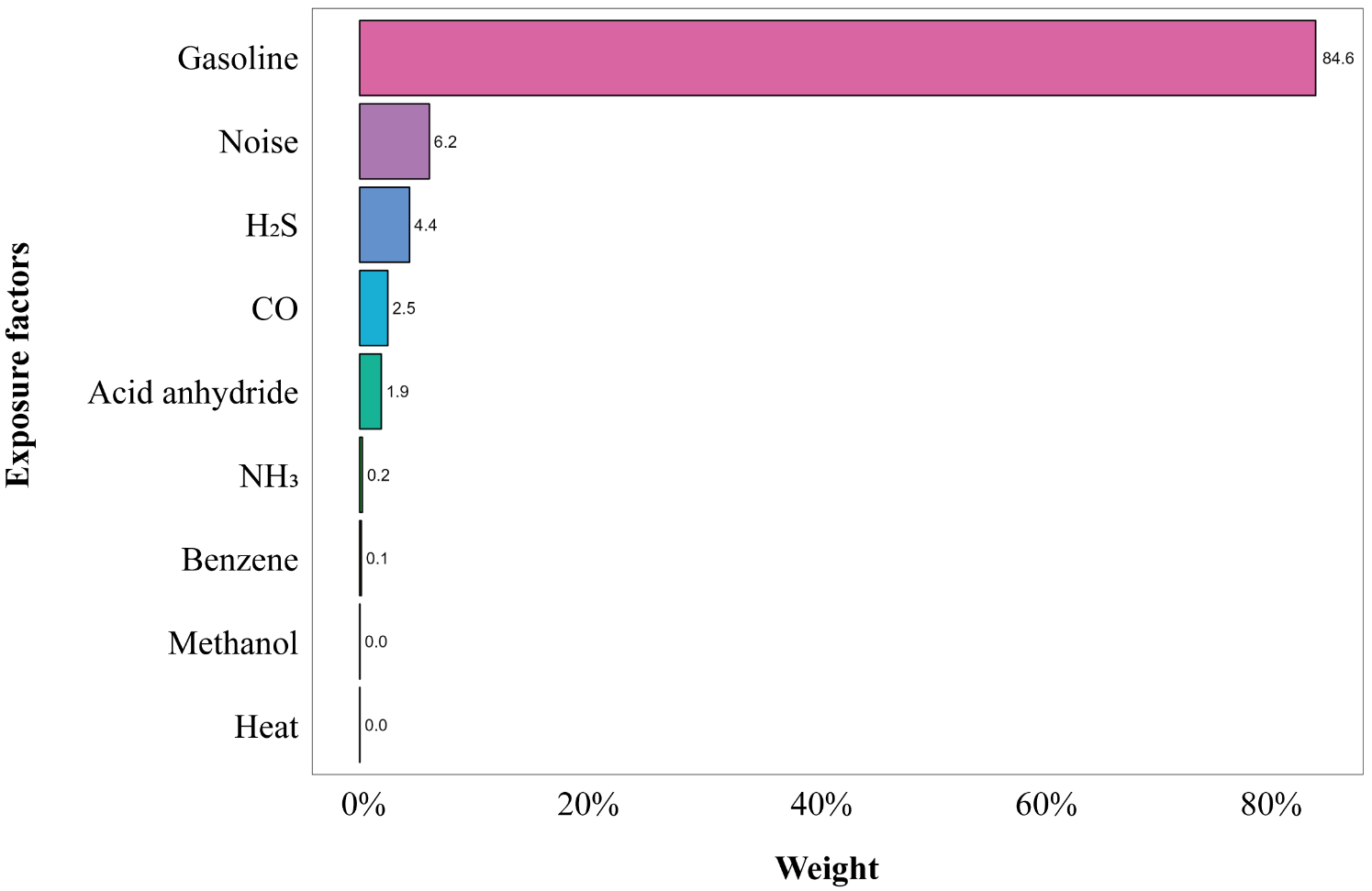


Figure S14 Negative-direction analysis of WQS model

2.2 Sensitivity analysis of WQS model

To further test the robustness of the results, we varied the train/validation split and random seed in the WQS model. Across all specifications, heat consistently contributed most to the overall effect, as shown in Figures S15-18.


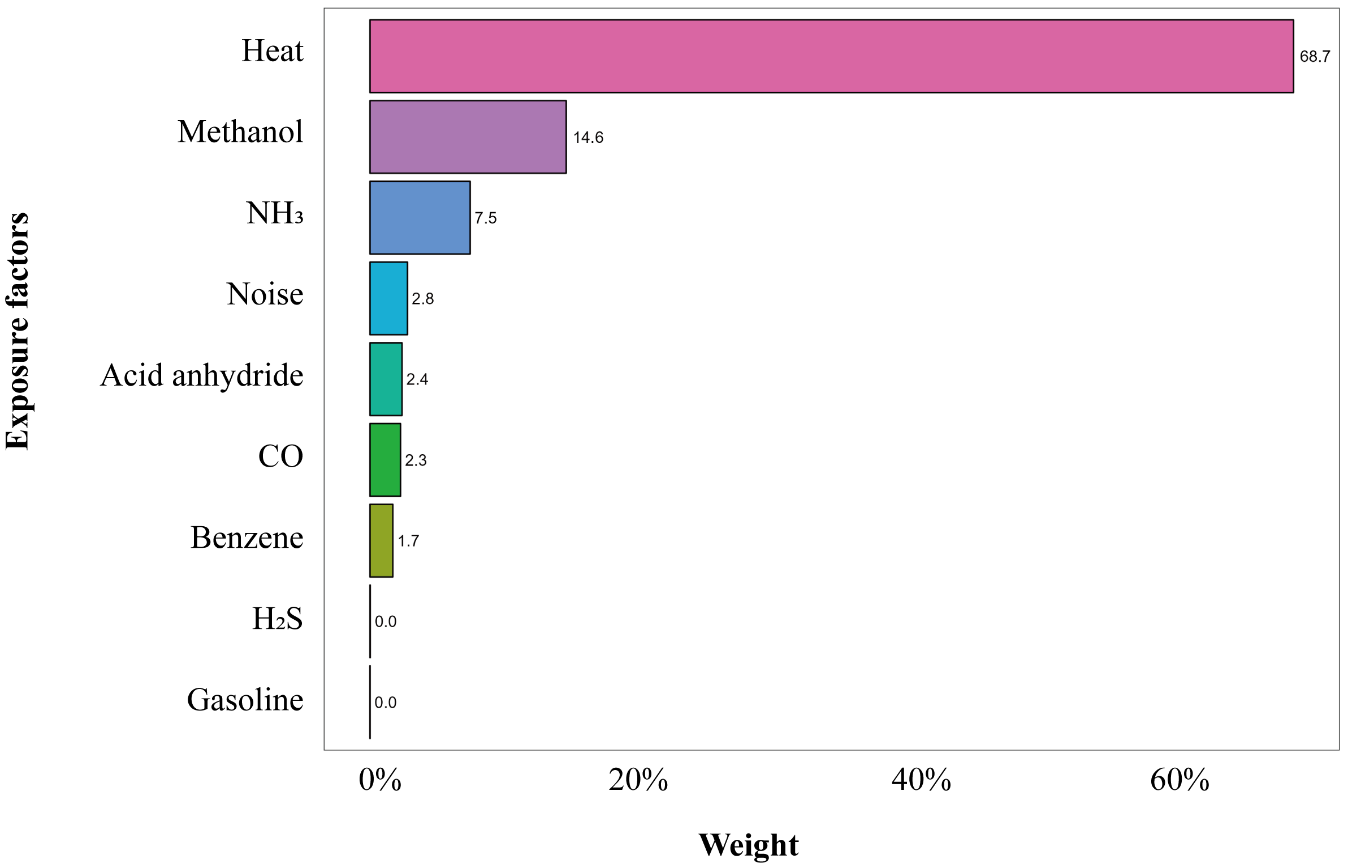


Figure S15 Sensitivity analysis of the WQS model (train/validation split 5:5, random seed =1800)


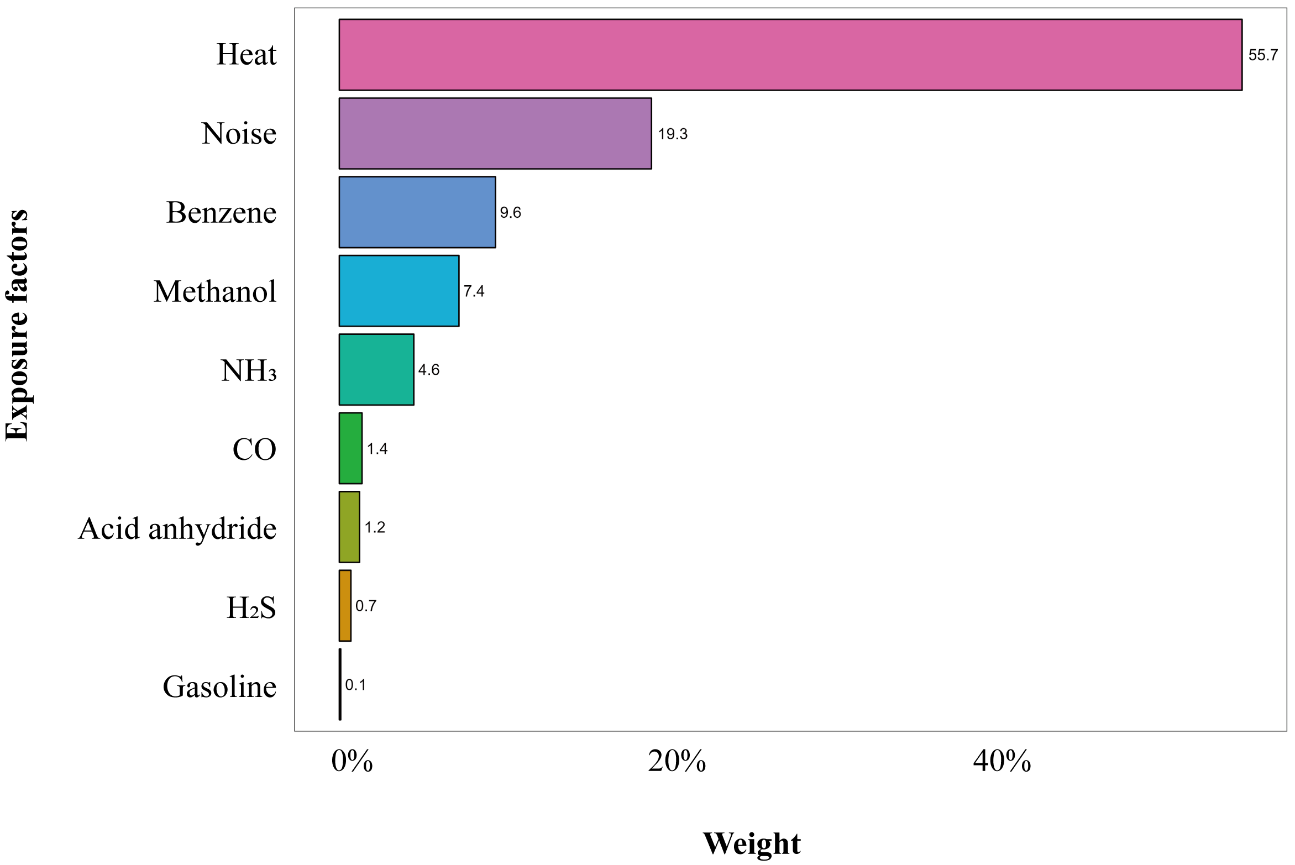


Figure S16 Sensitivity analysis of the WQS model (train/validation split 7:3, random seed=1800)


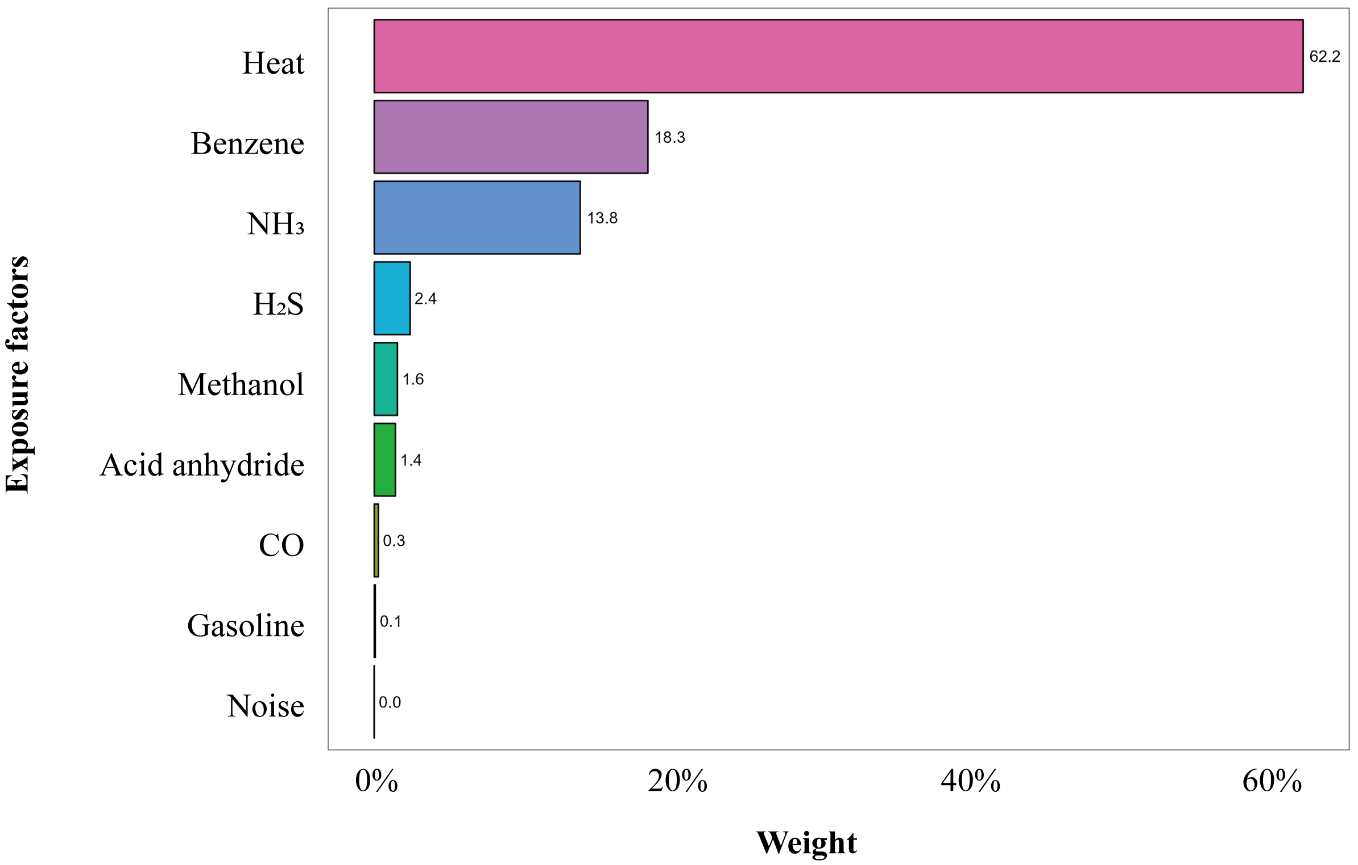


Figure S17 Sensitivity analysis of the WQS model (train/validation split 7:3, random seed=2000)


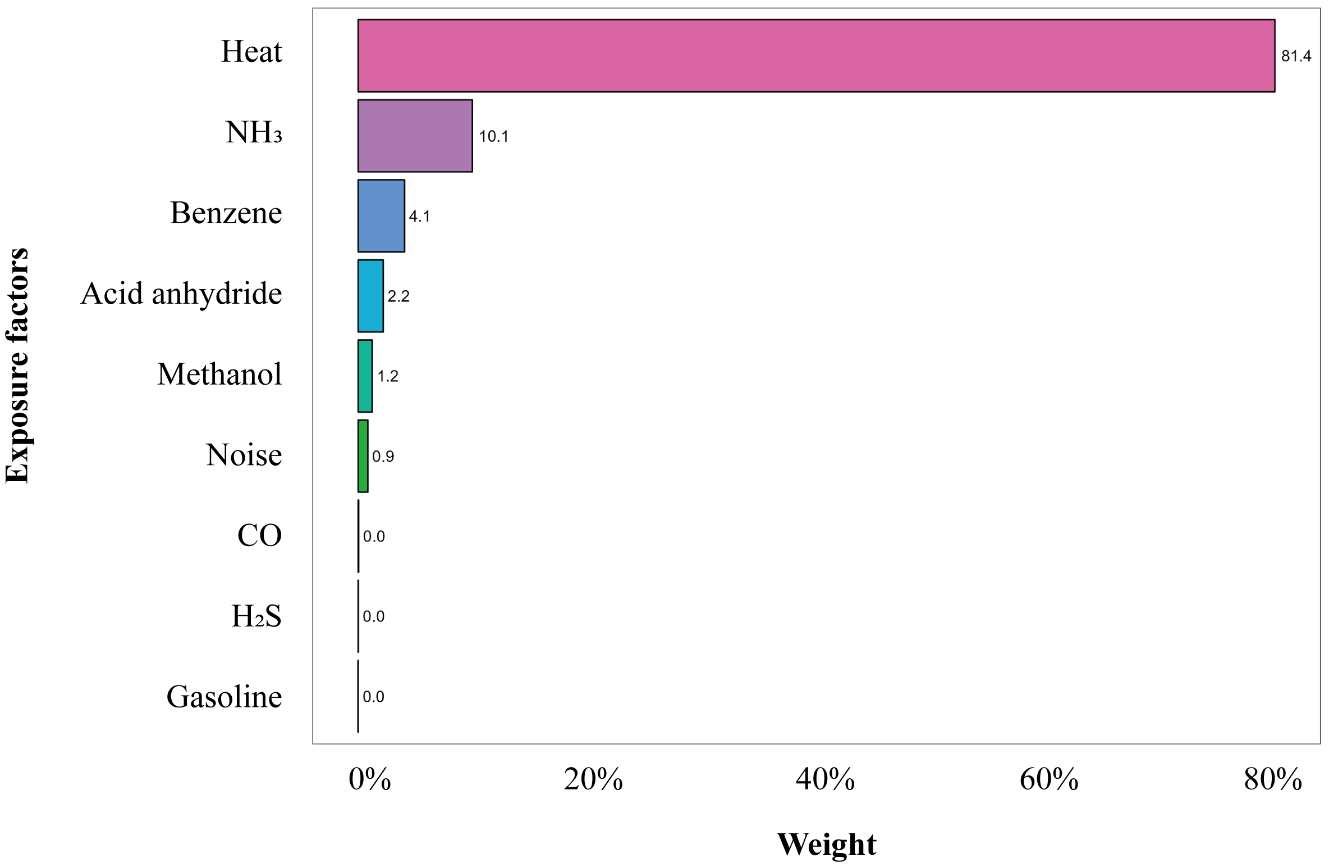


Figure S18 Sensitivity analysis of the WQS model (train/validation split 5:5, random seed=2000)

**3. Supplementary analysis of machine learning**

3.1 Dataset splitting for the development of a hyperuricemia risk prediction model using machine learning algorithms

To develop the hyperuricemia risk prediction model using machine learning algorithms, the dataset was split into a training set (n=1618) and a validation set (n=694) at a 7:3 ratio. The distributions of main covariates between the training and validation sets were balanced, ensuring good comparability. The details were shown in Table S4.

Table S4 Equilibrium test of training and validation sets [n (%) or X̅±SD]

| Variable | Total  (n=2312) | Validation set  (n=694) | Training set  (n=1618) | χ²/ t | *P* |
| --- | --- | --- | --- | --- | --- |
|  |  |  |  |  |  |
| BMI (kg/m^2^) | 23.73 ± 3.12 | 23.87 ± 3.26 | 23.67 ± 3.06 | 1.39 | 0.165 |
| Age (years) | 40.74 ± 11.21 | 40.57 ± 11.44 | 40.81 ± 11.11 | 0.47 | 0.638 |
| Length of service (years) | 20.05 ± 12.04 | 19.98 ± 12.32 | 20.09 ± 11.93 | 0.20 | 0.841 |
| Gender |  |  |  |  |  |
| Female | 667 (28.85) | 205 (29.54) | 462 (28.55) | 0.23 | 0.632 |
| Male | 1645 (71.15) | 489 (70.46) | 1156 (71.45) |  |  |
| Smoking |  |  |  |  |  |
| Never | 1602 (69.29) | 494 (71.18) | 1108 (68.48) | 2.41 | 0.300 |
| Occasionally | 263 (11.38) | 69 (9.94) | 194 (11.99) |  |  |
| Often | 447 (19.33) | 131 (18.88) | 316 (19.53) |  |  |
| Drinking |  |  |  |  |  |
| Never | 1226 (53.03) | 381 (54.90) | 845 (52.22) | 1.62 | 0.444 |
| Occasionally | 889 (38.45) | 259 (37.32) | 630 (38.94) |  |  |
| Often | 197 (8.52) | 54 (7.78) | 143 (8.84) |  |  |
| Heat |  |  |  |  |  |
| No | 1834 (79.33) | 549 (79.11) | 1285 (79.42) | 0.03 | 0.865 |
| Yes | 478 (20.67) | 145 (20.89) | 333 (20.58) |  |  |
| NH_3_ |  |  |  |  |  |
| No | 2018 (87.28) | 603 (86.89) | 1415 (87.45) | 0.14 | 0.708 |
| Yes | 294 (12.72) | 91 (13.11) | 203 (12.55) |  |  |
| Benzene |  |  |  |  |  |
| No | 1880 (81.31) | 579 (83.43) | 1301 (80.41) | 2.92 | 0.088 |
| Yes | 432 (18.69) | 115 (16.57) | 317 (19.59) |  |  |
| Methanol |  |  |  |  |  |
| No | 1958 (84.69) | 593 (85.45) | 1365 (84.36) | 0.44 | 0.507 |
| Yes | 354 (15.31) | 101 (14.55) | 253 (15.64) |  |  |
| H_2_S |  |  |  |  |  |
| No | 1519 (65.70) | 446 (64.27) | 1073 (66.32) | 0.91 | 0.341 |
| Yes | 793 (34.30) | 248 (35.73) | 545 (33.68) |  |  |
| Gasoline |  |  |  |  |  |
| No | 1616 (69.90) | 486 (70.03) | 1130 (69.84) | 0.01 | 0.927 |
| Yes | 696 (30.10) | 208 (29.97) | 488 (30.16) |  |  |
| Acid anhydrides |  |  |  |  |  |
| No | 1969 (85.16) | 585 (84.29) | 1384 (85.54) | 0.59 | 0.441 |
| Yes | 343 (14.84) | 109 (15.71) | 234 (14.46) |  |  |
| CO |  |  |  |  |  |
| No | 1872 (80.97) | 570 (82.13) | 1302 (80.47) | 0.87 | 0.351 |
| Yes | 440 (19.03) | 124 (17.87) | 316 (19.53) |  |  |
| Noise |  |  |  |  |  |
| No | 1351 (58.43) | 384 (55.33) | 967 (59.77) | 3.93 | 0.047 |
| Yes | 961 (41.57) | 310 (44.67) | 651 (40.23) |  |  |
| Hyperglycemia |  |  |  |  |  |
| No | 2284 (98.79) | 685 (98.70) | 1599 (98.83) | 0.06 | 0.805 |
| Yes | 28 (1.21) | 9 (1.30) | 19 (1.17) |  |  |
| Hypertension |  |  |  |  |  |
| No | 2077 (89.84) | 621 (89.48) | 1456 (89.99) | 0.14 | 0.712 |
| Yes | 235 (10.16) | 73 (10.52) | 162 (10.01) |  |  |

Note: t: t-test, χ²: Chi-square test; SD: standard deviation.

3.2 Demographic characteristics analysis of hyperuricemia in petrochemical workers (Training set)

In the training set of 1618 cases, petrochemical workers were divided into a hyperuricemia group (n=981) and control group (n=637). The results indicated that variables including BMI, age, length of service, smoking frequency, alcohol consumption, and occupational exposure factors (e.g., heat, NH_3_, benzene, noise, and CO) were significantly associated with hyperuricemia (*P*<0.05), as shown in Table S5.

Table S5 Basic characteristics and difference analysis of training set [n (%) or X̅±SD]

| Variable | Total  (n=1618) | Control group  (n=637) | hyperuricemia group (n=981) | χ²/ *t* | *P* |
| --- | --- | --- | --- | --- | --- |
|  |  |  |  |  |  |
| BMI (kg/m^2^) | 23.67 ± 3.06 | 23.01 ± 3.02 | 24.10 ± 3.02 | 7.07 | <0.001 |
| Age (years) | 40.81 ± 11.11 | 44.50 ± 11.07 | 38.42 ± 10.47 | 11.15 | <0.001 |
| Length of service (years) | 20.09 ± 11.93 | 23.19 ± 12.21 | 18.07 ± 11.30 | 8.64 | <0.001 |
| Gender |  |  |  |  |  |
| Female | 462 (28.55) | 301 (47.25) | 161 (16.41) | 180.07 | <0.001 |
| Male | 1156 (71.45) | 336 (52.75) | 820 (83.59) |  |  |
| Smoking |  |  |  |  |  |
| Never | 1108 (68.48) | 486 (76.30) | 622 (63.40) | 37.00 | <0.001 |
| Occasionally | 194 (11.99) | 43 (6.75) | 151 (15.39) |  |  |
| Often | 316 (19.53) | 108 (16.95) | 208 (21.20) |  |  |
| Drinking |  |  |  |  |  |
| Never | 845 (52.22) | 395 (62.01) | 450 (45.87) | 50.99 | <0.001 |
| Occasionally | 630 (38.94) | 180 (28.26) | 450 (45.87) |  |  |
| Often | 143 (8.84) | 62 (9.73) | 81 (8.26) |  |  |
| Heat |  |  |  |  |  |
| No | 1285 (79.42) | 564 (88.54) | 721 (73.50) | 53.47 | <0.001 |
| Yes | 333 (20.58) | 73 (11.46) | 260 (26.50) |  |  |
| NH_3_ |  |  |  |  |  |
| No | 1415 (87.45) | 596 (93.56) | 819 (83.49) | 35.75 | <0.001 |
| Yes | 203 (12.55) | 41 (6.44) | 162 (16.51) |  |  |
| Benzene |  |  |  |  |  |
| No | 1301 (80.41) | 544 (85.40) | 757 (77.17) | 16.62 | <0.001 |
| Yes | 317 (19.59) | 93 (14.60) | 224 (22.83) |  |  |
| Methanol |  |  |  |  |  |
| No | 1365 (84.36) | 565 (88.70) | 800 (81.55) | 14.96 | <0.001 |
| Yes | 253 (15.64) | 72 (11.30) | 181 (18.45) |  |  |
| H_2_S |  |  |  |  |  |
| No | 1073 (66.32) | 430 (67.50) | 643 (65.55) | 0.66 | 0.415 |
| Yes | 545 (33.68) | 207 (32.50) | 338 (34.45) |  |  |
| Gasoline |  |  |  |  |  |
| No | 1130 (69.84) | 448 (70.33) | 682 (69.52) | 0.12 | 0.729 |
| Yes | 488 (30.16) | 189 (29.67) | 299 (30.48) |  |  |
| Acid anhydrides |  |  |  |  |  |
| No | 1384 (85.54) | 578 (90.74) | 806 (82.16) | 22.97 | <0.001 |
| Yes | 234 (14.46) | 59 (9.26) | 175 (17.84) |  |  |
| CO |  |  |  |  |  |
| No | 1302 (80.47) | 545 (85.56) | 757 (77.17) | 17.30 | <0.001 |
| Yes | 316 (19.53) | 92 (14.44) | 224 (22.83) |  |  |
| Noise |  |  |  |  |  |
| No | 967 (59.77) | 407 (63.89) | 560 (57.08) | 7.45 | 0.006 |
| Yes | 651 (40.23) | 230 (36.11) | 421 (42.92) |  |  |
| Hyperglycemia |  |  |  |  |  |
| No | 1599 (98.83) | 628 (98.59) | 971 (98.98) | 0.52 | 0.473 |
| Yes | 19 (1.17) | 9 (1.41) | 10 (1.02) |  |  |
| Hypertension |  |  |  |  |  |
| No | 1456 (89.99) | 571 (89.64) | 885 (90.21) | 0.14 | 0.707 |
| Yes | 162 (10.01) | 66 (10.36) | 96 (9.79) |  |  |

Note: t: t-test, χ²: Chi-square test; SD: standard deviation

3.3 Feature selection using LASSO regression

Variables that showed significant differences in the training set were included and further analyzed using LASSO regression to identify key features influencing hyperuricemia. The results indicated that the LASSO regression model selected 17 non-zero coefficient variables, as shown in Table S6.

Table S6 Lasso coefficient table

| Variable | The minimum mean square error coefficient | The coefficient of the minimum distance standard error |
| --- | --- | --- |
| (Intercept) | 1.28 | -0.57 |
| BMI | 0.12 | 0.11 |
| Age | -0.16 | -0.06 |
| Gender | 1.48 | 1.32 |
| Smoking | 0.09 | 0.03 |
| Drinking | -0.21 | -0.08 |
| Length of service | 0.1 | 0.01 |
| NH_3_ | 0.22 | 0.0 |
| Benzene | 0.30 | 0.11 |
| Heat | 0.71 | 0.50 |
| Methanol | 0.0 | 0.0 |
| H_2_S | -0.15 | 0.0 |
| Gasoline | -0.64 | -0.48 |
| Acid anhydrides | -0.21 | 0.0 |
| CO | -0.03 | 0.0 |
| Noise | -0.19 | -0.06 |
| Hyperglycemia | -0.05 | 0.0 |
| Hypertension | -0.04 | 0.0 |

## 3.4 Model development and performance evaluation

(1) The specific fitting parameters for each model are detailed below:

Logistic Regression (C=: 1.0; max_iter: 100; penalty: l2; tol: 0.0001);

XGBClassifier (objective: binary:logistic);

LGBMClassifier (boosting_type: gbdt; learning_rate: 0.1; max_depth: -1; n_estimators: 100; num_leaves: 31);

RandomForestClassifier: (criterion: gini; max_depth: None; min_impurity_decrease: 0.0; n_estimators: 2);

AdaBoostClassifier: (learning_rate: 1.0; n_estimators: 50);

GaussianNB: (priors: None; var_smoothing: 1e-09);

ComplementNB: (alpha (Laplace/Lidstone): 1.0);

MLPClassifier: (activation: relu; hidden_layer_sizes: (20, 10); max_iter: 20);

SVC: (C: 1.0; kernel: rbf; tol: 0.001).

(2) Comparison of evaluation metrics for nine models in the training set

This study summarized the evaluation metrics of nine different models on both the training and validation sets, including AUC (Area Under the Curve), accuracy, sensitivity (recall), specificity, positive predictive value, negative predictive value, F1 score, and Kappa value. The XGBoost model showed the best performance on the training set, with an AUC of 1.00 and an accuracy of 0.97. However, it may exhibit significant bias on the validation set. In contrast, the LightGBM model demonstrated a high AUC of 0.98 and an accuracy of 0.93, suggesting better potential generalization ability. The details are shown in Table S7.

Table S7 Comparison of model evaluation indicators (training set)

| Models | AUC | Cutoff | Accuracy | Sensitivity | Specificity | Positive predictive value | Negative predictive value | F1 score | Kappa |
| --- | --- | --- | --- | --- | --- | --- | --- | --- | --- |
| XGBoost | 1.00 | 0.54 | 0.97 | 0.97 | 0.98 | 0.98 | 0.96 | 0.98 | 0.94 |
| logistic | 0.78 | 0.57 | 0.73 | 0.78 | 0.65 | 0.77 | 0.67 | 0.78 | 0.43 |
| LightGBM | 0.98 | 0.53 | 0.93 | 0.94 | 0.92 | 0.95 | 0.91 | 0.94 | 0.85 |
| RandomForest | 1.00 | 0.55 | 0.99 | 0.99 | 1.00 | 1.00 | 0.99 | 1.00 | 0.99 |
| AdaBoost | 0.89 | 0.50 | 0.80 | 0.80 | 0.82 | 0.869 | 0.73 | 0.83 | 0.60 |
| GNB | 0.74 | 0.47 | 0.71 | 0.76 | 0.62 | 0.75 | 0.64 | 0.76 | 0.38 |
| CNB | 0.71 | 0.21 | 0.71 | 0.79 | 0.59 | 0.744 | 0.65 | 0.77 | 0.39 |
| MLP | 0.79 | 0.58 | 0.73 | 0.77 | 0.66 | 0.773 | 0.66 | 0.77 | 0.43 |
| SVM | 0.77 | 0.59 | 0.72 | 0.78 | 0.64 | 0.764 | 0.66 | 0.77 | 0.42 |

(3) Comparison of evaluation metrics for nine models in the validation set

On the validation set, the LightGBM model demonstrated the best performance, with an AUC of 0.87 and an accuracy of 0.79. Both sensitivity and specificity were relatively high, indicating stable performance on unseen data and a lower risk of overfitting compared to XGBoost. Although the RandomForest model performed exceptionally well on the training set (AUC=1.00), its AUC decreased to 0.85 on the validation set, suggesting potential overfitting. The details are shown in Table S8.

Table S8 Comparison of evaluation metrics for the nine models on the validation set

| Models | AUC | Cutoff | Accuracy | Sensitivity | Specificity | Positive predictive value | Negative predictive value | F1 score | Kappa |
| --- | --- | --- | --- | --- | --- | --- | --- | --- | --- |
| XGBoost | 0.86 | 0.54 | 0.78 | 0.81 | 0.74 | 0.83 | 0.72 | 0.82 | 0.55 |
| logistic | 0.78 | 0.57 | 0.72 | 0.77 | 0.64 | 0.77 | 0.66 | 0.77 | 0.41 |
| LightGBM | 0.87 | 0.53 | 0.79 | 0.81 | 0.77 | 0.84 | 0.73 | 0.83 | 0.57 |
| RandomForest | 0.85 | 0.55 | 0.79 | 0.82 | 0.74 | 0.83 | 0.73 | 0.82 | 0.55 |
| AdaBoost | 0.87 | 0.50 | 0.78 | 0.78 | 0.79 | 0.85 | 0.71 | 0.81 | 0.56 |
| GNB | 0.73 | 0.47 | 0.69 | 0.76 | 0.60 | 0.74 | 0.62 | 0.75 | 0.36 |
| CNB | 0.71 | 0.21 | 0.71 | 0.79 | 0.58 | 0.74 | 0.65 | 0.77 | 0.38 |
| MLP | 0.78 | 0.58 | 0.72 | 0.77 | 0.65 | 0.77 | 0.65 | 0.77 | 0.41 |
| SVM | 0.77 | 0.59 | 0.72 | 0.78 | 0.63 | 0.76 | 0.65 | 0.77 | 0.40 |

(4) Model development and performance evaluation

This study established a hyperuricemia risk prediction model using nine supervised machine learning (ML) algorithms. The ROC curve results for the training set showed that the Random Forest model had the highest AUC (1.00), followed by XGBoost model (0.99) and Logistic Regression (0.98). In the validation set, AUC values generally decreased by approximately 0.05–0.08, with LightGBM and AdaBoost exhibiting the great performance, both AUC (0.87).

The calibration curves show that the LightGBM model achieved the best calibration performance, with a Brier score of 0.145 (95% CI: 0.139–0.151), closely following the ideal diagonal line. Random Forest (0.155) and XGBoost (0.157) also demonstrated good calibration reliability. In contrast, AdaBoost, GaussianNB, and ComplementNB exhibited poor calibration, with substantial deviations from the perfectly calibrated line, indicating over- or underestimation of event probabilities.

As shown in the validation DCA, the LightGBM, XGBoost, and Random Forest models consistently provided higher net benefits than the “Treat All” and “Treat None” strategies across most threshold probabilities (10%–80%), indicating superior clinical applicability. Logistic regression and SVM achieved moderate net benefits, whereas AdaBoost, GaussianNB, ComplementNB, and MLP showed limited or no advantage over the default strategies. Overall, the LightGBM model demonstrated the highest net benefit and potential clinical utility for hyperuricemia risk prediction.

The precision–recall (PR) curves of nine machine learning models in the training dataset show that tree-based ensemble models demonstrated superior performance compared with linear and probabilistic classifiers. Random Forest achieved the highest average precision (AP = 1.0, 95% CI: 0.1–1.0), followed by XGBoost (AP = 0.998, 95% CI: 0.997–0.999) and LightGBM (AP = 0.988, 95% CI: 0.987–0.989). These models exhibited near-perfect discrimination, suggesting excellent fit but also indicating potential overfitting. AdaBoost showed moderately high performance (AP = 0.925, 95% CI: 0.921–0.923), while the multilayer perceptron (AP = 0.834, 95% CI: 0.830–0.836) and support vector classifier (AP = 0.825, 95% CI: 0.823–0.827) achieved relatively balanced precision–recall trade-offs. Logistic regression yielded an AP of 0.831 (95% CI: 0.828–0.834), serving as a stable baseline. In contrast, the Gaussian and Complement Naïve Bayes models showed lower performance (AP = 0.788 and 0.752, respectively). Collectively, these results indicate that ensemble-based models, particularly LightGBM, provide the best predictive ability for hyperuricemia while maintaining a favorable balance between precision and recall. The details are shown in Figure S19.


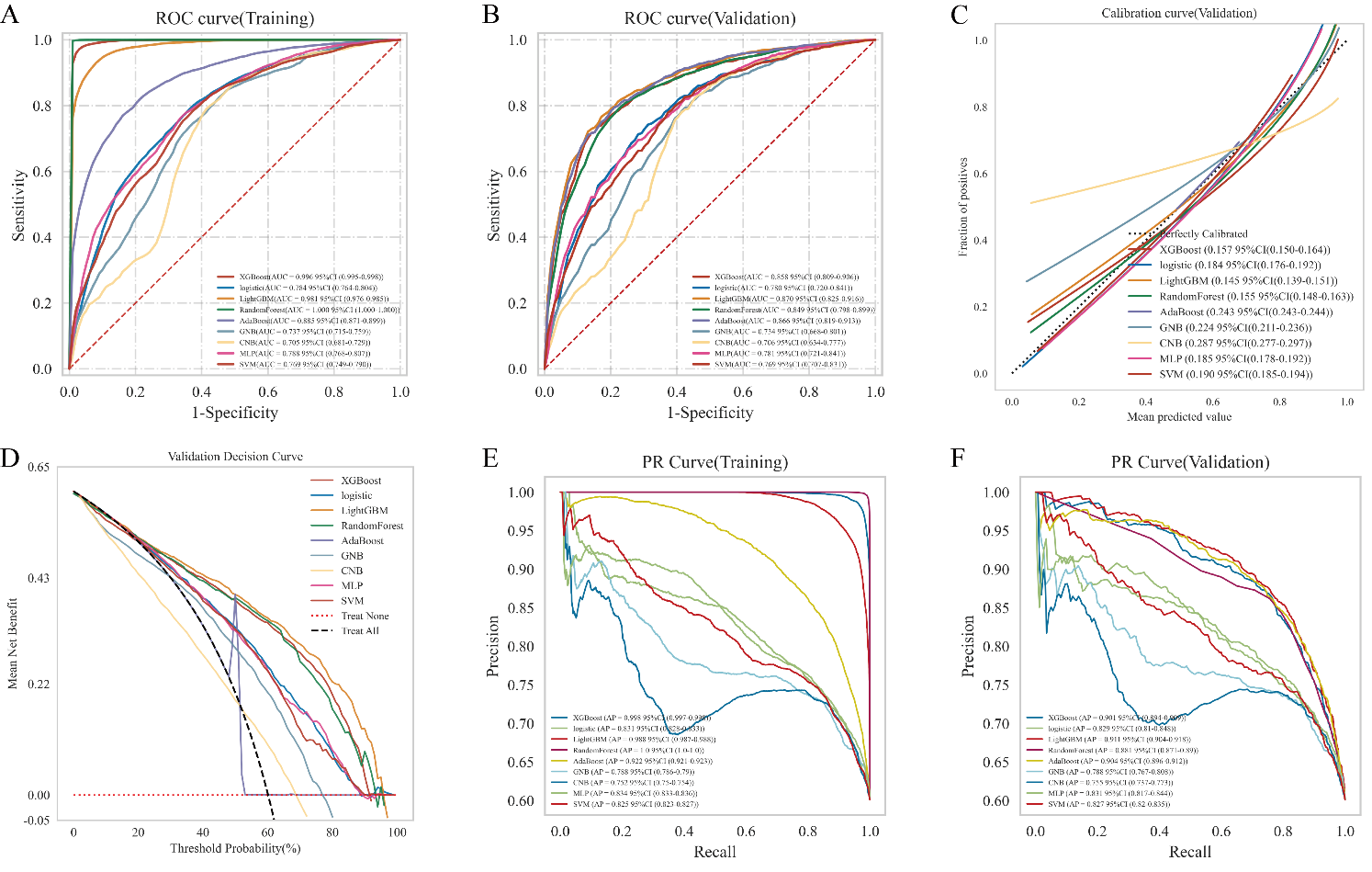


Figure S19 Consolidated performance evaluation of nine machine learning models.

Note: A and B represent ROC curves for the training and validation sets, respectively; C and D represent precision rate-recall curves for the training and validation sets, respectively; E represents the validation set calibration curve; and F represents the validation set decision curve.

(5) Comparison of Delong Test Z-values for the nine models

This study evaluated the differences in predictive performance among the nine models using Delong test Z-values. A smaller Z-value indicates a smaller difference between the two models, while a larger Z-value indicates a greater difference. The results showed that the differences between XGBoost, LightGBM, and Random Forest were relatively small. The details are shown in Table S9 and Figure S20.

Table S9 Mean Z-values from the delong test for the nine models

| Models | XGBoost | LR | LGBM | RF | AdaBoost | GBDT | GNB | MLP | SVM |
| --- | --- | --- | --- | --- | --- | --- | --- | --- | --- |
| XGBoost | NA | 2.7 | 1.55 | 1.15 | 0.59 | 3.71 | 4.14 | 2.67 | 2.97 |
| LR | 2.7 | NA | 3.30 | 2.43 | 3.46 | 2.13 | 2.41 | 0.92 | 1.01 |
| LGBM | 1.55 | 3.30 | NA | 1.67 | 0.38 | 4.21 | 4.65 | 3.29 | 3.57 |
| RF | 1.15 | 2.43 | 1.67 | NA | 1.07 | 3.48 | 4.02 | 2.42 | 2.73 |
| AdaBoost | 0.59 | 3.46 | 0.38 | 1.07 | NA | 4.43 | 4.73 | 3.37 | 3.60 |
| GBDT | 3.71 | 2.13 | 4.21 | 3.48 | 4.43 | NA | 1.43 | 1.72 | 1.41 |
| GNB | 4.14 | 2.41 | 4.65 | 4.02 | 4.73 | 1.43 | NA | 2.7 | 2.83 |
| MLP | 2.67 | 0.92 | 3.29 | 2.42 | 3.37 | 1.72 | 2.7 | NA | 0.76 |
| SVM | 2.97 | 1.01 | 3.57 | 2.73 | 3.60 | 1.41 | 2.83 | 0.76 | NA |


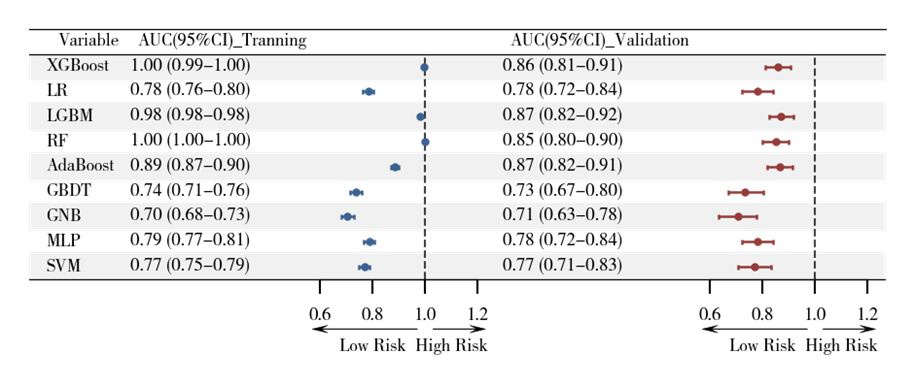


Figure S20 Mean of cross-validated receiver operating characteristic curves with 10 folds for the nine models.

3.5 LightGBM model

The performance metrics of the LightGBM model, including the AUC, optimal cutoff value, and accuracy across the training, validation, and test sets are summarized in the table below (Table S10).

Table S10 Evaluation metrics of the model on the training, validation and test sets

| AUC | Cutoff | Accuracy | Sensitivity | Specificity | Positive predictive value | Negative predictive value | F1 score |
| --- | --- | --- | --- | --- | --- | --- | --- |
| Training set | | | | | | | |
| 0.99 (0.01) | 0.53(0.02) | 0.95(0.01) | 0.95(0.01) | 0.96(0.01) | 0.97(0.01) | 0.93(0.01) | 0.96 (0.01) |
| Validation set | | | | | | | |
| 0.86 (0.03) | 0.53 (0.03) | 0.78 (0.03) | 0.81 (0.03) | 0.75(0.05) | 0.83 (0.03) | 0.73 (0.04) | 0.82(0.03) |
| Test set | | | | | | | |
| 0.86 | 0.49 | 0.79 | 0.85 | 0.71 | 0.82 | 0.75 | 0.84 |

3.6 Sensitivity analysis of the LightGBM model

To assess transportability, we have re-split the dataset for training and test, in which the training dataset primarily included data from 2013–2019 and the test dataset included data from 2020–2022. This approach ensured that the model was trained in earlier years and tested in later years.

Apart from the dataset split, the included variables and analytical methods were identical to those described in the main text. The model parameters were as follows: boosting_type = gbdt; colsample_bytree = 1.0; learning_rate = 0.1; max_depth =–1; min_child_samples = 20; n_estimators = 100; num_leaves = 31; reg_alpha = 0.0; reg_lambda = 0.0; subsample = 1.0.

(1) LightGBM model training and evaluation

In the training set, the LightGBM model exhibited great performance with an AUC of 0.99, accuracy of 0.97 (0.00), sensitivity (0.96 ± 0.01) and specificity (0.97 ± 0.01). These metrics indicate a strong fit to the training data but also suggest potential overfitting. In the validation set, the model maintained good discrimination (AUC = 0.88 ± 0.02) and balanced classification performance (accuracy = 0.81 ± 0.02), the sensitivity (0.83 ± 0.03) and specificity (0.77 ± 0.04) were well-balanced. In the test set, the model achieved an AUC of 0.82, with a cutoff of 0.54 and accuracy of 0.73. Sensitivity (0.68) and specificity (0.81) were moderately balanced. Nevertheless, The F1-score of 0.75 supports an overall satisfactory balance between precision and recall. The learning curve of the LGBMClassifier demonstrates high training performance but limited generalization to the validation set. The calibration plot indicates systematic bias in the predicted probabilities, suggesting that post-processing calibration is necessary before practical application. Decision curve analysis reveals that the model provides the greatest clinical net benefit in the low-to-medium threshold probability range, supporting its utility for guiding interventions in moderate-risk populations. Furthermore, the confusion matrix suggests potential overfitting on the training set, highlighting the need for external validation in future studies. Finally, the KS plot confirms excellent discriminative ability (KS = 0.502) between positive and negative classes, with optimal separation achieved at a decision threshold of 0.624. The details are shown in Table S11 and Figure S21.


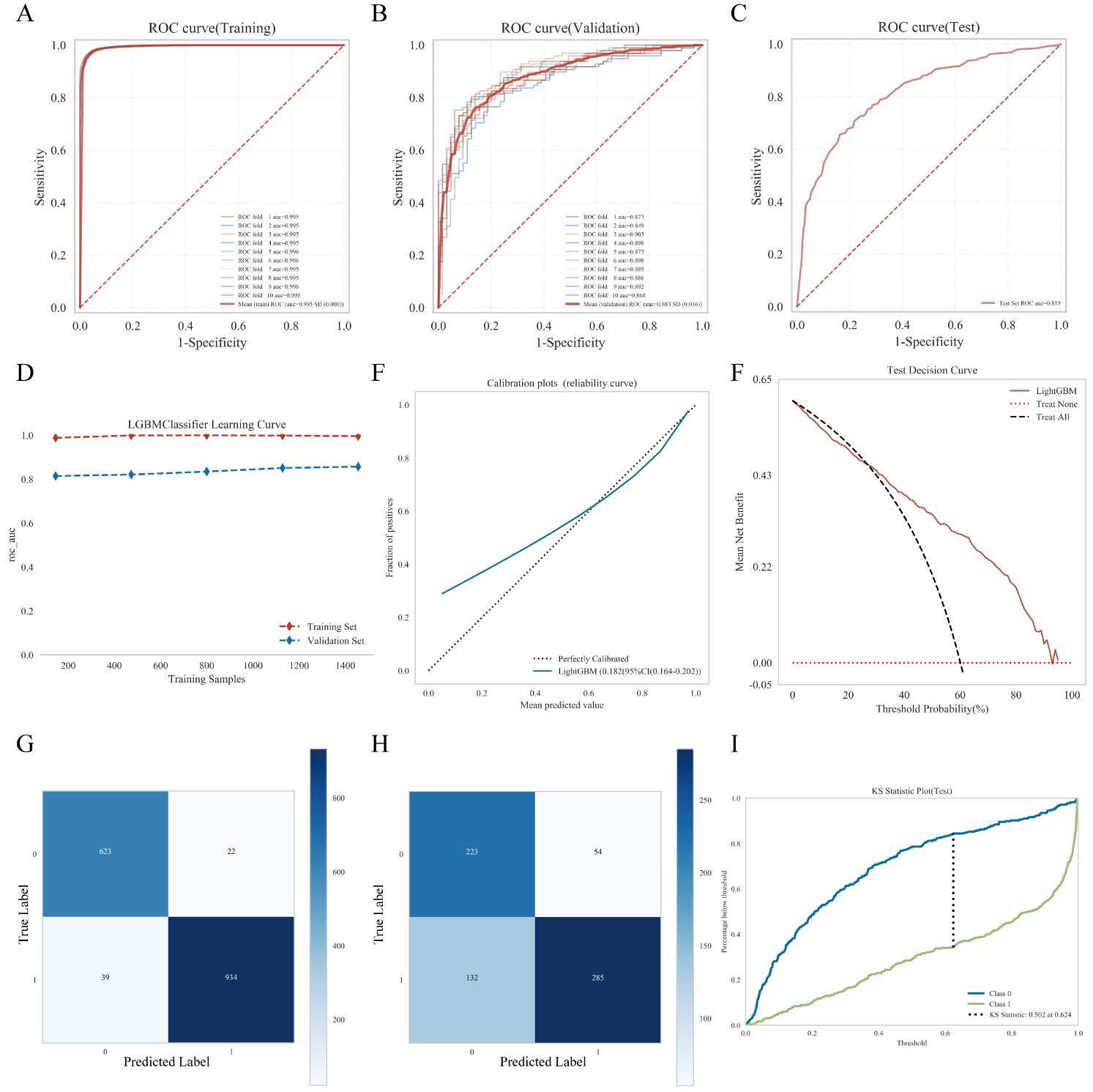


Figure S21 Consolidated performance evaluation of the LightGBM machine learning model.

Note: A is ROC curve (Training set); B is ROC curve (Validation set); C is ROC curve (Test set); D is learning curve; E is calibration plots; F is test decision curve; G is confusion matrix of training set; H is confusion matrix of test set; and I is KS statistical diagram

Table S11 Evaluation metrics of the model on the training, validation and test set (95%CI)

| AUC | Cutoff | Accuracy | Sensitivity | Specificity | Positive predictive value | Negative predictive value | F1 score |
| --- | --- | --- | --- | --- | --- | --- | --- |
| Training set | | | | | | | |
| 0.99 (0.00) | 0.53 (0.03) | 0.97 (0.00) | 0.96 (0.01) | 0.97 (0.01) | 0.98 (0.00) | 0.95 (0.00) | 0.97(0.01) |
| Validation set | | | | | | | |
| 0.88 (0.02) | 0.53 (0.03) | 0.81 (0.02) | 0.83 (0.03) | 0.77 (0.04) | 0.85 (0.02) | 0.75 (0.02) | 0.84 (0.01) |
| Test set | | | | | | | |
| 0.82 | 0.54 | 0.73 | 0.68 | 0.81 | 0.84 | 0.63 | 0.75 |

(2) Quantifying the contribution of predictive features using SHAP values

SHAP analysis revealed that length of service, age, BMI, gender and heat exposure, and length of service were among the most influential predictors. Higher BMI and heat were associated with an increased risk of hyperuricemia, consistent with known epidemiological evidence. The details are shown in Figure S22.


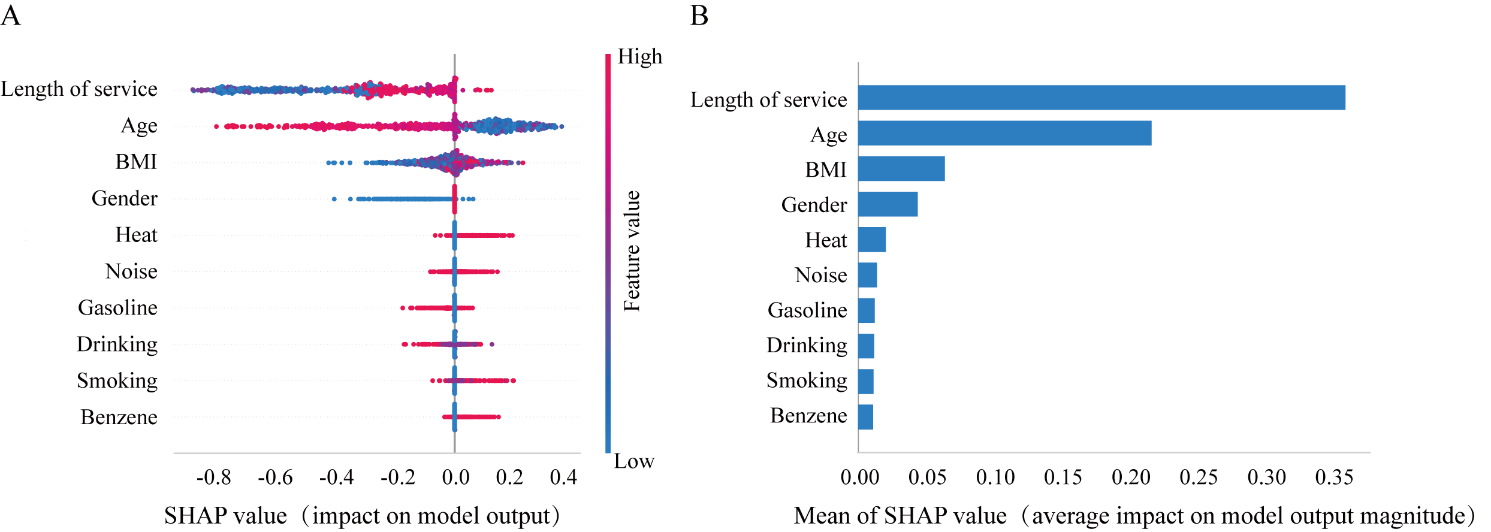


Figure S22 SHAP values quantify the contribution of predicted features.

Note: A is the summary of SHAP values for variables in the LightGBM Model. The horizontal axis represents the contribution of each feature to the model, and the vertical axis ranks the features based on the total sum of their SHAP values. The color indicates the feature value, with red corresponding to high values and blue corresponding to low values; and B is the histogram of feature importance for the LightGBM Model.

Overall, after re-splitting the dataset into training and test sets, the LightGBM model also demonstrated strong performance on the training set, however, its generalizability to the test set was limited. The calibration plot, decision curve analysis, and KS statistic yielded results consistent with those presented in the main text, suggesting reasonable model stability. In the final SHAP analysis, the top five contributing features were length of service, age, BMI, gender and heat exposure. Among occupational hazards, heat exposure remained positively associated with an increased risk of hyperuricemia, in line with the findings reported in the main analysis.

**4. TRIPOD Checklist: Prediction Model Development**

Version: 11-January-2024

| **Section/Topic** | **Item** | **Development / evaluation**1 | **Checklist item** | **Reported on page** |
| --- | --- | --- | --- | --- |
| **TITLE** | | | |  |
| *Title* | 1 | D;E | Identify the study as developing or evaluating the performance of a multivariable prediction model, the target population, and the outcome to be predicted | 1 |
| **ABSTRACT** | | | | |
| *Abstract* | 2 | D;E | See TRIPOD+AI for Abstracts checklist | 1-2 |
| **INTRODUCTION** | | | | |
| *Background* | 3a | D;E | Explain the healthcare context (including whether diagnostic or prognostic) and rationale for developing or evaluating the prediction model, including references to existing models | Not applicable |
|  | 3b | D;E | Describe the target population and the intended purpose of the prediction model in the context of the care pathway, including its intended users (e.g., healthcare professionals, patients, public) | 2-3 |
|  | 3c | D;E | Describe any known health inequalities between sociodemographic groups | 2-3 |
| *Objectives* | 4 | D;E | Specify the study objectives, including whether the study describes the development or validation of a prediction model (or both) | 3 |
| **METHODS** | | | | |
| *Data* | 5a | D;E | Describe the sources of data separately for the development and evaluation datasets (e.g., randomised trial, cohort, routine care or registry data), the rationale for using these data, and representativeness of the data | 3 |
|  | 5b | D;E | Specify the dates of the collected participant data, including start and end of participant accrual; and, if applicable, end of follow-up | 3 |
| *Participants* | 6a | D;E | Specify key elements of the study setting (e.g., primary care, secondary care, general population) including the number and location of centres | 3 |
|  | 6b | D;E | Describe the eligibility criteria for study participants | 4 |
|  | 6c | D;E | Give details of any treatments received, and how they were handled during model development or evaluation, if relevant | Not applicable |
| *Data preparation* | 7 | D;E | Describe any data pre-processing and quality checking, including whether this was similar across relevant sociodemographic groups | 3-4 |
| *Outcome* | 8a | D;E | Clearly define the outcome that is being predicted and the time horizon, including how and when assessed, the rationale for choosing this outcome, and whether the method of outcome assessment is consistent across sociodemographic groups | 4 |
|  | 8b | D;E | If outcome assessment requires subjective interpretation, describe the qualifications and demographic characteristics of the outcome assessors | Not applicable |
|  | 8c | D;E | Report any actions to blind assessment of the outcome to be predicted | Not applicable |
| *Predictors* | 9a | D | Describe the choice of initial predictors (e.g., literature, previous models, all available predictors) and any pre-selection of predictors before model building | Not applicable |
|  | 9b | D;E | Clearly define all predictors, including how and when they were measured (and any actions to blind assessment of predictors for the outcome and other predictors) | 4-5 |
|  | 9c | D;E | If predictor measurement requires subjective interpretation, describe the qualifications and demographic characteristics of the predictor assessors | Not applicable |
| *Sample size* | 10 | D;E | Explain how the study size was arrived at (separately for development and evaluation), and justify that the study size was sufficient to answer the research question. Include details of any sample size calculation | 4-5 |
| *Missing data* | 11 | D;E | Describe how missing data were handled. Provide reasons for omitting any data | 5 |
| *Analytical methods* | 12a | D | Describe how the data were used (e.g., for development and evaluation of model performance) in the analysis, including whether the data were partitioned, considering any sample size requirements | 5-6 |
|  | 12b | D | Depending on the type of model, describe how predictors were handled in the analyses (functional form, rescaling, transformation, or any standardisation). | Not applicable |
|  | 12c | D | Specify the type of model, rationale^2^, all model-building steps, including any hyperparameter tuning, and method for internal validation | 5-6 and Supplementary 21-22 |
|  | 12d | D;E | Describe if and how any heterogeneity in estimates of model parameter values and model performance was handled and quantified across clusters (e.g., hospitals, countries). See TRIPOD-Cluster for additional considerations^3^ | Not applicable |
|  | 12e | D;E | Specify all measures and plots used (and their rationale) to evaluate model performance (e.g., discrimination, calibration, clinical utility) and, if relevant, to compare multiple models | 5-6 and Supplementary 22-25 |
|  | 12f | E | Describe any model updating (e.g., recalibration) arising from the model evaluation, either overall or for particular sociodemographic groups or settings | Not applicable |
|  | 12g | E | For model evaluation, describe how the model predictions were calculated (e.g., formula, code, object, application programming interface) | Not applicable |
| *Class imbalance* | 13 | D;E | If class imbalance methods were used, state why and how this was done, and any subsequent methods to recalibrate the model or the model predictions | Not applicable |
| *Fairness* | 14 | D;E | Describe any approaches that were used to address model fairness and their rationale | Not applicable |
| *Model output* | 15 | D | Specify the output of the prediction model (e.g., probabilities, classification). Provide details and rationale for any classification and how the thresholds were identified | Not applicable |
| *Training versus*  *evaluation* | 16 | D;E | Identify any differences between the development and evaluation data in healthcare setting, eligibility criteria, outcome, and predictors | Not applicable |
| *Ethical approval* | 17 | D;E | Name the institutional research board or ethics committee that approved the study and describe the participant-informed consent or the ethics committee waiver of informed consent | 4 |
| **OPEN SCIENCE** | | | | |
| *Funding* | 18a | D;E | Give the source of funding and the role of the funders for the present study | Not applicable |
| *Conflicts of*  *interest* | 18b | D;E | Declare any conflicts of interest and financial disclosures for all authors | 11 |
| *Protocol* | 18c | D;E | Indicate where the study protocol can be accessed or state that a protocol was not prepared | Not applicable |
| *Registration* | 18d | D;E | Provide registration information for the study, including register name and registration number, or state that the study was not registered | Not applicable |
| *Data sharing* | 18e | D;E | Provide details of the availability of the study data | 12 |
| *Code sharing* | 18f | D;E | Provide details of the availability of the analytical code4 | Supplmmentary 35-59 |
| **PATIENT & PUBLIC INVOLVEMENT** | | | | |
| *Patient & Public Involvement* | 19 | D;E | Provide details of any patient and public involvement during the design, conduct, reporting, interpretation, or dissemination of the study or state no involvement. | Not applicable |
| **RESULTS** | | | | |
| *Participants* | 20a | D;E | Describe the flow of participants through the study, including the number of participants with and without the outcome and, if applicable, a summary of the follow-up time. A diagram may be helpful. | 6 |
|  | 20b | D;E | Report the characteristics overall and, where applicable, for each data source or setting, including the key dates, key predictors (including demographics), treatments received, sample size, number of  outcome events, follow-up time, and amount of missing data. A table may be helpful. Report any differences across key demographic groups. | 6 and 19-20 |
|  | 20c | E | For model evaluation, show a comparison with the development data of the distribution of important predictors (demographics, predictors, and outcome). | Not applicable |
| *Model development* | 21 | D;E | Specify the number of participants and outcome events in each analysis (e.g., for model development, hyperparameter tuning, model evaluation) | Supplmmentary 17-20 |
| *Model*  *specification* | 22 | D | Provide details of the full prediction model (e.g., formula, code, object, application programming interface) to allow predictions in new individuals and to enable third-party evaluation and implementation, including any restrictions to access or re-use (e.g., freely available, proprietary)5 | 7 and Supplmmentary 17-20 |
| *Model*  *performance* | 23a | D;E | Report model performance estimates with confidence intervals, including for any key subgroups (e.g., sociodemographic). Consider plots to aid presentation. | Supplmmentary 20-25 |
|  | 23b | D;E | If examined, report results of any heterogeneity in model performance across clusters. See TRIPOD Cluster for additional details3. | Not applicable |
| *Model updating* | 24 | E | Report the results from any model updating, including the updated model and subsequent performance | Not applicable |
| **DISCUSSION** | | | | |
| *Interpretation* | 25 | D;E | Give an overall interpretation of the main results, including issues of fairness in the context of the objectives and previous studies | 7-8 |
| *Limitations* | 26 | D;E | Discuss any limitations of the study (such as a non-representative sample, sample size, overfitting, missing data) and their effects on any biases, statistical uncertainty, and generalizability | 10-11 |
| *Usability of the*  *model in the*  *context of current care* | 27a | D | Describe how poor quality or unavailable input data (e.g., predictor values) should be assessed and handled when implementing the prediction model | Not applicable |
|  | 27b | D | Specify whether users will be required to interact in the handling of the input data or use of the model, and what level of expertise is required of users | Not applicable |
|  | 27c | D;E | Discuss any next steps for future research, with a specific view to applicability and generalizability of the model | 11 |

Note:

1 D=items relevant only to the development of a prediction model; E=items relating solely to the evaluation of a prediction model; D;E=items applicable

to both the development and evaluation of a prediction model

2 Separately for all model building approaches.

3 TRIPOD-Cluster is a checklist of reporting recommendations for studies developing or validating models that explicitly account for clustering or explore

heterogeneity in model performance (eg, at different hospitals or centres). Debray et al, BMJ 2023; 380: e071018 [DOI: 10.1136/bmj-2022-071018]

4This relates to the analysis code, for example, any data cleaning, feature engineering, model building, evaluation.

5 This relates to the code to implement the model to get estimates of risk for a new individual.

**5. Analysis code**

5.1 Three-stage stepwise adjusted GLM models code as follow:

install.packages("flextable")

install.packages("officer")

install.packages("openxlsx")

install.packages("interactionR")

library(interactionR)

library(flextable)

library(officer)

library(openxlsx)

library(readxl)

data<- read_excel("data.xlsx")

cols_to_factor <- c(7:9, 11:22)

data[cols_to_factor] <- lapply(data[cols_to_factor], factor)

y <- as.numeric(data$HYPERURICEMIA)

fit1<-glm(HYPERURICEMIA~x+covariate, data=data,family=binomial())

## Note: Fit models sequentially for each variable and covariate adjustment.

## variable (x): Heat, NH3, Benzene, Methanol, H2S, Gasoline, Acid_Anhydride, CO, and Noise.

## Covariate adjustment:

## - Model 1: Unadjusted

## - Model 2: Adjusted for Gender, Age, and BMI

## - Model 3: Further adjusted for Gender, Age, BMI, Length of service, Smoking, Drinking, Hyperglycemia, and Hypertension.

coef <- summary(fit1)$coefficients

p_values <- as.numeric(coef[, "Pr(>|z|)"])

results <- data.frame(

Variable = rownames(coef),

OR = exp(coef[, "Estimate"]),

`Lower 95% CI` = exp(coef[, "Estimate"] - 1.96 * coef[, "Std. Error"]),

`Upper 95% CI` = exp(coef[, "Estimate"] + 1.96 * coef[, "Std. Error"]),

`P-value` = p_values

)

results$Significance <- cut(

results$`P.value`,

breaks = c(-Inf, 0.001, 0.01, 0.05, 0.1, Inf),

labels = c("***", "**", "*", ".", " "),

right = FALSE

)

desktop_path <- file.path(Sys.getenv("USERPROFILE"), "Desktop", "results.xlsx")

write.xlsx(results, desktop_path, rowNames = FALSE)

5.2 WQS regression code as follow:

library(readxl)

library(dplyr)

library(tidyr)

library(gWQS)

library(glmnet)

library(ggplot2)

library(knitr)

library(kableExtra)

library(reshape2)

library(openxlsx)

y <- as.numeric(data$HYPERURICEMIA)

X <- as.matrix(data[, c(7:9, 11:22)])

exposure <- colnames(data[11:19])

WQS <- gwqs(HYPERURICEMIA~ wqs

+BMI +Length of service+ Age+ Gender+ Smoking + Drinking,

mix_name = exposure,

data = data,

q = NULL,

validation = 0.6,

b = 200,

b1_pos = TRUE,

b_constr = FALSE,

family = "binomial",

signal = "expt",

seed = 1800,

plots = TRUE,

tables = TRUE)

coef_original <- coef(WQS)

confint_original <- confint(WQS)

summary_WQS <- summary(WQS)

p_values <- summary_WQS$coefficients[, "Pr(>|z|)"]

result <- cbind(coef_original, confint_original, p_values)

colnames(result) <- c("Coefficient", "2.5% CI", "97.5% CI", "P-value")

print(result)

coef_exp <- exp(coef(WQS))

confint_exp <- exp(confint(WQS))

summary_WQS <- summary(WQS)

p_values <- summary_WQS$coefficients[, "Pr(>|z|)"]

result <- cbind(coef_exp, confint_exp, p_values)

colnames(result) <- c("Exp(Coefficient)", "2.5% CI", "97.5% CI", "P-value")

print(result)

gwqs_weights_tab(WQS)

library(ggplot2)

gwqs_barplot(WQS)+

theme(panel.grid.major = element_blank(),

panel.grid.minor = element_blank(),

panel.background = element_blank(),

axis.line = element_line())

options(digits = 3)

gwqs_barplot(WQS)

w_ord <- order(WQS$final_weights$mean_weight)

mean_weight <- WQS$final_weights$mean_weight[w_ord]

mix_name <- factor(WQS$final_weights$mix_name[w_ord],

levels = WQS$final_weights$mix_name[w_ord])

dataplot <- data.frame(mean_weight, mix_name)

library(ggplot2)

plot <- ggplot(dataplot, aes(x = mix_name, y = mean_weight, fill = mix_name))+

geom_bar(stat = "identity", color = "black")+

geom_text(aes(label = sprintf("%.1f", mean_weight * 100)), hjust = -0.2, size = 4) +

theme_bw() +

theme(

axis.ticks = element_blank(),

axis.text.x = element_text(color = 'black', family = "Times New Roman", size = 14),

axis.text.y = element_text(family = "Times New Roman", size = 14),

axis.title.x = element_text(size = 16, family = "Times New Roman", margin = margin(t = 10)),

axis.title.y = element_text(size = 16, family = "Times New Roman", margin = margin(r = 10)),

legend.position = "none",

panel.grid = element_blank()

) +

scale_y_continuous(

labels = function(x) sprintf("%.0f%%", x * 100)

) + coord_flip() +

xlab("Exposure factors hyperuricemia ") +

ylab("Weight")

ggsave("tiff", plot = plot, width = 12, height = 8)

5.3 Adjust heat as a covariate into the models of chemical exposures to examine the association between occupational hazards and hyperuricemia

library(interactionR)

library(flextable)

library(officer)

library(openxlsx)

library(readxl)

data<- read_excel("data.xlsx")

fit1<-glm(HYPERURICEMIA ~x+covariate, data=data,family=binomial())

## Note: Fit models sequentially for each variable and covariate adjustment.

## variable (x): NH3, Benzene, Methanol, H2S, Gasoline, Acid_Anhydride, CO, and Noise.

## Covariate adjustment:

## - Model 1: Adjusted for Heat

## - Model 2: Adjusted for Heat, Gender, Age, and BMI

## - Model 3: Further adjusted for Heat, Gender, Age, BMI, Length of service, Smoking, Drinking, Hyperglycemia, and Hypertension.

coef <- summary(fit1)$coefficients

p_values <- as.numeric(coef[, "Pr(>|z|)"])

results <- data.frame(

Variable = rownames(coef),

OR = exp(coef[, "Estimate"]),

`Lower 95% CI` = exp(coef[, "Estimate"] - 1.96 * coef[, "Std. Error"]),

`Upper 95% CI` = exp(coef[, "Estimate"] + 1.96 * coef[, "Std. Error"]),

`P-value` = p_values

)

results$Significance <- cut(

results$`P.value`,

breaks = c(-Inf, 0.001, 0.01, 0.05, 0.1, Inf),

labels = c("***", "**", "*", ".", " "),

right = FALSE

)

save_path <- file.path(Sys.getenv("USERPROFILE"), "Desktop", "results.xlsx")

write_xlsx(results, path = save_path)

5.4 Additive interaction analysis of heat exposure and other occupational hazards

library(readxl)

library(openxlsx)

library(writexl)

data<- read_excel("data.xlsx")

data$Heat_bin <- as.numeric(as.character(data$Heat))

data$Noise_bin <- as.numeric(as.character(data$Noise))

data$NH3_bin <- as.numeric(as.character(data$NH3))

data$Benzene_bin <- as.numeric(as.character(data$Benzene))

data$Methanol_bin <- as.numeric(as.character(data$Methanol))

data$H2S_bin <- as.numeric(as.character(data$H2S))

data$Gasoline_bin <- as.numeric(as.character(data$Gasoline))

data$Acid_Anhydride_bin <- as.numeric(as.character(data$Acid_Anhydride))

data$CO_bin <- as.numeric(as.character(data$CO))

data$HYPERURICEMIA_bin <- as.numeric(as.character(data$HYPERURICEMIA))

fit1<-glm(HYPERURICEMIA_bin~Heat_bin* x

+ Age+ Gender+BMI +Length of service+ Smoking + Drinking+ Hyperglycemia+ Hyperglycemia

,data=data,family=binomial())

## Note: Fit models sequentially for each variable and covariate adjustment.

## variable (x): NH3_bin, Benzene_bin, Methanol_bin, H2S_bin, Gasoline_bin, Acid_Anhydride_bin, CO_bin, and Noise_bin.

out<- interactionR(fit1,

exposure_names =c("Heat_bin", "Noise_bin"),

ci.type = "mover",

ci.level = 0.95,

em = FALSE,

recode = TRUE)

out1<-out$dframe

save_path <- file.path("D: "result.xlsx")

write_xlsx(out1, path = save_path)

5.5 Analysis code of subgroup analysis of GLM investigate the association between occupational hazard exposure and hyperuricemia

5.5.1 Sex-stratified analysis.

library(dplyr)

library(openxlsx)

library(readxl)

data <- data %>%

exposure_vars <- c("Heat_bin", "NH3_bin", "Benzene_bin", "Methanol_bin", "H2S_bin", "Gasoline_bin", "Acid_Anhydride_bin", "CO_bin", "Noise_bin")

subgroup_results <- list()

for (group in unique(data$Gender)) {

cat(group, "\n")

data_sub <- data %>% filter(Gender == group)

group_result <- data.frame()

for (var in exposure_vars) {

formula_str <- paste0("HYPERURICEMIA_bin ~ ", var, "covariate")

## Note: Fit models sequentially for covariate adjustment.

## Covariate adjustment:

## - Model 1: Unadjusted

## - Model 2: Adjusted for Gender, Age, and BMI

## - Model 3: Further adjusted for Gender, Age, BMI, Length of service, Smoking, Drinking, Hyperglycemia, and Hypertension.

## In the sex-stratified analysis. Model 2: Adjusted for Age and BMI; Model 3: Further adjusted for Age, BMI, Length of service, Smoking, Drinking, Hyperglycemia, and Hypertension.

## In the age-stratified analysis. Model 2: Adjusted for Gender and BMI; Model 3: Further adjusted for Gender, BMI, Length of service, Smoking, Drinking, Hyperglycemia, and Hypertension.

## In the BMI-stratified analysis. Model 2: Adjusted for Age and Gender; Model 3: Further adjusted for Age, Gender, Length of service, Smoking, Drinking, Hyperglycemia, and Hypertension.

fit <- glm(as.formula(formula_str), data = data_sub, family = binomial())

coef_table <- summary(fit)$coefficients

row_name <- grep(paste0("^", var), rownames(coef_table), value = TRUE)

if (length(row_name) == 1) {

est <- coef_table[row_name, "Estimate"]

se <- coef_table[row_name, "Std. Error"]

pval <- coef_table[row_name, "Pr(>|z|)"]

or <- exp(est)

ci_low <- exp(est - 1.96 * se)

ci_high <- exp(est + 1.96 * se)

sig <- cut(pval,

breaks = c(-Inf, 0.001, 0.01, 0.05, 0.1, Inf),

labels = c("***", "**", "*", ".", " "),

right = FALSE)

group_result <- rbind(group_result, data.frame(

Gender = group,

Variable = var,

OR = round(or, 2),

`Lower 95% CI` = round(ci_low, 2),

`Upper 95% CI` = round(ci_high, 2),

`P-value` = signif(pval, 3),

Significance = sig

))

}

}

subgroup_results[[as.character(group)]] <- group_result

}

final_results <- do.call(rbind, subgroup_results)

write.xlsx(final_results, file = "C:\\ Desktop\\ results.xlsx", rowNames = FALSE)

5.5.2 Age-stratified analysis

data <- data %>%

mutate(Age_group = case_when(

Age < 30 ~ "<30",

Age >= 30 & Age <= 45 ~ "30-45",

Age > 45 ~ ">45"

))

exposure_vars <- c("Heat_bin", "NH3_bin", "Benzene_bin", "Methanol_bin", "H2S_bin", "Gasoline_bin", "Acid_Anhydride_bin", "CO_bin", "Noise_bin")

subgroup_results <- list()

for (group in unique(data$Age_group)) {

cat(group, "\n")

data_sub <- data %>% filter(Age_group == group)

group_result <- data.frame()

for (var in exposure_vars) {

formula_str <- paste0("HYPERURICEMIA_bin ~ ", var, "covariate")

## Note: Fit models sequentially for covariate adjustment.

## Covariate adjustment:

## - Model 1: Unadjusted

## - Model 2: Adjusted for Gender, Age, and BMI

## - Model 3: Further adjusted for Gender, Age, BMI, Length of service, Smoking, Drinking, Hyperglycemia, and Hypertension.

## In the sex-stratified analysis. Model 2: Adjusted for Age and BMI; Model 3: Further adjusted for Age, BMI, Length of service, Smoking, Drinking, Hyperglycemia, and Hypertension.

## In the age-stratified analysis. Model 2: Adjusted for Gender and BMI; Model 3: Further adjusted for Gender, BMI, Length of service, Smoking, Drinking, Hyperglycemia, and Hypertension.

## In the BMI-stratified analysis. Model 2: Adjusted for Age and Gender; Model 3: Further adjusted for Age, Gender, Length of service, Smoking, Drinking, Hyperglycemia, and Hypertension.

fit <- glm(as.formula(formula_str), data = data_sub, family = binomial())

coef_table <- summary(fit)$coefficients

row_name <- grep(paste0("^", var), rownames(coef_table), value = TRUE)

if (length(row_name) == 1) {

est <- coef_table[row_name, "Estimate"]

se <- coef_table[row_name, "Std. Error"]

pval <- coef_table[row_name, "Pr(>|z|)"]

or <- exp(est)

ci_low <- exp(est - 1.96 * se)

ci_high <- exp(est + 1.96 * se)

sig <- cut(pval,

breaks = c(-Inf, 0.001, 0.01, 0.05, 0.1, Inf),

labels = c("***", "**", "*", ".", " "),

right = FALSE)

group_result <- rbind(group_result, data.frame(

Gender = group,

Variable = var,

OR = round(or, 2),

`Lower 95% CI` = round(ci_low, 2),

`Upper 95% CI` = round(ci_high, 2),

`P-value` = signif(pval, 3),

Significance = sig

))

}

}

subgroup_results[[as.character(group)]] <- group_result

}

final_results <- do.call(rbind, subgroup_results)

write.xlsx(final_results, file = "C:\\ Desktop\\ results.xlsx", rowNames = FALSE)

5.5.3 BMI-stratified analysis.

data <- data %>%

mutate(BMI_group = case_when(

BMI < 24 ~ "<24",

BMI >= 24 & BMI <= 28 ~ "24-28",

BMI > 28 ~ ">28"

))

exposure_vars <- c("Heat_bin", "NH3_bin", "Benzene_bin", "Methanol_bin", "H2S_bin", "Gasoline_bin", "Acid_Anhydride_bin", "CO_bin", "Noise_bin")

subgroup_results <- list()

for (group in unique(data$BMI_group)) {

cat(group, "\n")

data_sub <- data %>% filter(BMI_group == group)

group_result <- data.frame()

for (var in exposure_vars) {

formula_str <- paste0("HYPERURICEMIA_bin ~ ", var, "covariate")

## Note: Fit models sequentially for covariate adjustment.

## Covariate adjustment:

## - Model 1: Unadjusted

## - Model 2: Adjusted for Gender, Age, and BMI

## - Model 3: Further adjusted for Gender, Age, BMI, Length of service, Smoking, Drinking, Hyperglycemia, and Hypertension.

## In the sex-stratified analysis. Model 2: Adjusted for Age and BMI; Model 3: Further adjusted for Age, BMI, Length of service, Smoking, Drinking, Hyperglycemia, and Hypertension.

## In the age-stratified analysis. Model 2: Adjusted for Gender and BMI; Model 3: Further adjusted for Gender, BMI, Length of service, Smoking, Drinking, Hyperglycemia, and Hypertension.

## In the BMI-stratified analysis. Model 2: Adjusted for Age and Gender; Model 3: Further adjusted for Age, Gender, Length of service, Smoking, Drinking, Hyperglycemia, and Hypertension.

fit <- glm(as.formula(formula_str), data = data_sub, family = binomial())

coef_table <- summary(fit)$coefficients

row_name <- grep(paste0("^", var), rownames(coef_table), value = TRUE)

if (length(row_name) == 1) {

est <- coef_table[row_name, "Estimate"]

se <- coef_table[row_name, "Std. Error"]

pval <- coef_table[row_name, "Pr(>|z|)"]

or <- exp(est)

ci_low <- exp(est - 1.96 * se)

ci_high <- exp(est + 1.96 * se)

sig <- cut(pval,

breaks = c(-Inf, 0.001, 0.01, 0.05, 0.1, Inf),

labels = c("***", "**", "*", ".", " "),

right = FALSE)

group_result <- rbind(group_result, data.frame(

Gender = group,

Variable = var,

OR = round(or, 2),

`Lower 95% CI` = round(ci_low, 2),

`Upper 95% CI` = round(ci_high, 2),

`P-value` = signif(pval, 3),

Significance = sig

))

}

}

subgroup_results[[as.character(group)]] <- group_result

}

final_results <- do.call(rbind, subgroup_results)

write.xlsx(final_results, file = "C:\\ Desktop\\ results.xlsx", rowNames = FALSE)

5.5.4 Length of service-stratified analysis.

data <- data %>%

mutate(Length of service_group = case_when(

Length of service <= 10 ~ "<=10",

Length of service > 10 ~ ">10"

))

exposure_vars <- c("Heat_bin", "NH3_bin", "Benzene_bin", "Methanol_bin", "H2S_bin", "Gasoline_bin", "Acid_Anhydride_bin", "CO_bin", "Noise_bin")

subgroup_results <- list()

for (group in unique(data$(Length of service_group)) {

cat(group, "\n")

data_sub <- data %>% filter((Length of service_group == group)

group_result <- data.frame()

for (var in exposure_vars) {

formula_str <- paste0("HYPERURICEMIA_bin ~ ", var, "covariate")

## Note: Fit models sequentially for covariate adjustment.

## Covariate adjustment:

## - Model 1: Unadjusted

## - Model 2: Adjusted for Gender, Age, and BMI

## - Model 3: Further adjusted for Gender, Age, BMI, Length of service, Smoking, Drinking, Hyperglycemia, and Hypertension.

## In the sex-stratified analysis. Model 2: Adjusted for Age and BMI; Model 3: Further adjusted for Age, BMI, Length of service, Smoking, Drinking, Hyperglycemia, and Hypertension.

## In the age-stratified analysis. Model 2: Adjusted for Gender and BMI; Model 3: Further adjusted for Gender, BMI, Length of service, Smoking, Drinking, Hyperglycemia, and Hypertension.

## In the BMI-stratified analysis. Model 2: Adjusted for Age and Gender; Model 3: Further adjusted for Age, Gender, Length of service, Smoking, Drinking, Hyperglycemia, and Hypertension.

fit <- glm(as.formula(formula_str), data = data_sub, family = binomial())

coef_table <- summary(fit)$coefficients

row_name <- grep(paste0("^", var), rownames(coef_table), value = TRUE)

if (length(row_name) == 1) {

est <- coef_table[row_name, "Estimate"]

se <- coef_table[row_name, "Std. Error"]

pval <- coef_table[row_name, "Pr(>|z|)"]

or <- exp(est)

ci_low <- exp(est - 1.96 * se)

ci_high <- exp(est + 1.96 * se)

sig <- cut(pval,

breaks = c(-Inf, 0.001, 0.01, 0.05, 0.1, Inf),

labels = c("***", "**", "*", ".", " "),

right = FALSE)

group_result <- rbind(group_result, data.frame(

Gender = group,

Variable = var,

OR = round(or, 2),

`Lower 95% CI` = round(ci_low, 2),

`Upper 95% CI` = round(ci_high, 2),

`P-value` = signif(pval, 3),

Significance = sig

))

}

}

subgroup_results[[as.character(group)]] <- group_result

}

final_results <- do.call(rbind, subgroup_results)

write.xlsx(final_results, file = "C:\\ Desktop\\ results.xlsx", rowNames = FALSE)

5.6 Negative-direction analysis of WQS model

WQS <- gwqs(HYPERURICEMIA~ wqs

+BMI +Length of service+ Age+ Gender+ Smoking + Drinking,

mix_name = exposure,

data = data,

q = NULL,

validation = 0.6,

b = 1000,

b1_pos = FALSE,

b_constr = TRUE,

family = "binomial",

signal = "expt",

seed = 1800,

plots = TRUE,

tables = TRUE)

coef_original <- coef(WQS)

confint_original <- confint(WQS)

summary_WQS <- summary(WQS)

p_values <- summary_WQS$coefficients[, "Pr(>|z|)"]

result <- cbind(coef_original, confint_original, p_values)

colnames(result) <- c("Coefficient", "2.5% CI", "97.5% CI", "P-value")

print(result)

coef_exp <- exp(coef(WQS))

confint_exp <- exp(confint(WQS))

summary_WQS <- summary(WQS)

p_values <- summary_WQS$coefficients[, "Pr(>|z|)"]

result <- cbind(coef_exp, confint_exp, p_values)

colnames(result) <- c("Exp(Coefficient)", "2.5% CI", "97.5% CI", "P-value")

print(result)

gwqs_weights_tab(WQS)

library(ggplot2)

gwqs_barplot(WQS)+

theme(panel.grid.major = element_blank(),

panel.grid.minor = element_blank(),

panel.background = element_blank(),

axis.line = element_line())

options(digits = 3)

gwqs_barplot(WQS)

w_ord <- order(WQS$final_weights$mean_weight)

mean_weight <- WQS$final_weights$mean_weight[w_ord]

mix_name <- factor(WQS$final_weights$mix_name[w_ord],

levels = WQS$final_weights$mix_name[w_ord])

dataplot <- data.frame(mean_weight, mix_name)

library(ggplot2)

plot <- ggplot(dataplot, aes(x = mix_name, y = mean_weight, fill = mix_name))+

geom_bar(stat = "identity", color = "black")+

geom_text(aes(label = sprintf("%.1f", mean_weight * 100)), hjust = -0.2, size = 4) +

theme_bw() +

theme(

axis.ticks = element_blank(),

axis.text.x = element_text(color = 'black', family = "Times New Roman", size = 14),

axis.text.y = element_text(family = "Times New Roman", size = 14),

axis.title.x = element_text(size = 16, family = "Times New Roman", margin = margin(t = 10)),

axis.title.y = element_text(size = 16, family = "Times New Roman", margin = margin(r = 10)),

legend.position = "none",

panel.grid = element_blank()

) +

scale_y_continuous(

labels = function(x) sprintf("%.0f%%", x * 100)

) + coord_flip() +

xlab("Exposure factors hyperuricemia ") +

ylab("Weight")

ggsave("tiff", plot = plot, width = 12, height = 8)

5.7 Sensitivity analysis of WQS model

##Sensitivity analysis of the WQS model (train/validation split 5:5, random seed =1800)

WQS <- gwqs(HYPERURICEMIA~ wqs

+ BMI +Length of service+ Age+ Gender+ Smoking + Drinking,

mix_name = exposure,

data = data,

q = NULL,

validation = 0.5,

b = 1000,

b1_pos = TRUE,

b_constr = FALSE,

family = "binomial",

signal = "expt",

seed = 1800,

plots = TRUE,

tables = TRUE)

coef_original <- coef(WQS)

confint_original <- confint(WQS)

summary_WQS <- summary(WQS)

p_values <- summary_WQS$coefficients[, "Pr(>|z|)"]

result <- cbind(coef_original, confint_original, p_values)

colnames(result) <- c("Coefficient", "2.5% CI", "97.5% CI", "P-value")

print(result)

gwqs_weights_tab(WQS)

library(ggplot2)

gwqs_barplot(WQS)+

theme(panel.grid.major = element_blank(),

panel.grid.minor = element_blank(),

panel.background = element_blank(),

axis.line = element_line())

options(digits = 3)

gwqs_barplot(WQS)

w_ord <- order(WQS$final_weights$mean_weight)

mean_weight <- WQS$final_weights$mean_weight[w_ord]

mix_name <- factor(WQS$final_weights$mix_name[w_ord],

levels = WQS$final_weights$mix_name[w_ord])

dataplot <- data.frame(mean_weight, mix_name)

library(ggplot2)

plot <- ggplot(dataplot, aes(x = mix_name, y = mean_weight, fill = mix_name))+

geom_bar(stat = "identity", color = "black")+

geom_text(aes(label = sprintf("%.1f", mean_weight * 100)), hjust = -0.2, size = 4) +

theme_bw() +

theme(

axis.ticks = element_blank(),

axis.text.x = element_text(color = 'black', family = "Times New Roman", size = 14),

axis.text.y = element_text(family = "Times New Roman", size = 14),

axis.title.x = element_text(size = 16, family = "Times New Roman", margin = margin(t = 10)),

axis.title.y = element_text(size = 16, family = "Times New Roman", margin = margin(r = 10)),

legend.position = "none",

panel.grid = element_blank()

) +

scale_y_continuous(

labels = function(x) sprintf("%.0f%%", x * 100)

) + coord_flip() +

xlab("Exposure factors hyperuricemia ") +

ylab("Weight")

ggsave("tiff", plot = plot, width = 12, height = 8)

##Sensitivity analysis of the WQS model (train/validation split 7:3, random seed=1800)

WQS <- gwqs(HYPERURICEMIA~ wqs

+BMI +Length of service+ Age+ Gender+ Smoking + Drinking,

mix_name = exposure,

data = data,

q = NULL,

validation = 0.7,

b = 1000,

b1_pos = TRUE,

b_constr = FALSE,

family = "binomial",

signal = "expt",

seed = 1800,

plots = TRUE,

tables = TRUE)

coef_original <- coef(WQS)

confint_original <- confint(WQS)

summary_WQS <- summary(WQS)

p_values <- summary_WQS$coefficients[, "Pr(>|z|)"]

result <- cbind(coef_original, confint_original, p_values)

colnames(result) <- c("Coefficient", "2.5% CI", "97.5% CI", "P-value")

print(result)

gwqs_weights_tab(WQS)

library(ggplot2)

gwqs_barplot(WQS)+

theme(panel.grid.major = element_blank(),

panel.grid.minor = element_blank(),

panel.background = element_blank(),

axis.line = element_line())

options(digits = 3)

gwqs_barplot(WQS)

w_ord <- order(WQS$final_weights$mean_weight)

mean_weight <- WQS$final_weights$mean_weight[w_ord]

mix_name <- factor(WQS$final_weights$mix_name[w_ord],

levels = WQS$final_weights$mix_name[w_ord])

dataplot <- data.frame(mean_weight, mix_name)

library(ggplot2)

plot <- ggplot(dataplot, aes(x = mix_name, y = mean_weight, fill = mix_name))+

geom_bar(stat = "identity", color = "black")+

geom_text(aes(label = sprintf("%.1f", mean_weight * 100)), hjust = -0.2, size = 4) +

theme_bw() +

theme(

axis.ticks = element_blank(),

axis.text.x = element_text(color = 'black', family = "Times New Roman", size = 14),

axis.text.y = element_text(family = "Times New Roman", size = 14),

axis.title.x = element_text(size = 16, family = "Times New Roman", margin = margin(t = 10)),

axis.title.y = element_text(size = 16, family = "Times New Roman", margin = margin(r = 10)),

legend.position = "none",

panel.grid = element_blank()

) +

scale_y_continuous(

labels = function(x) sprintf("%.0f%%", x * 100)

) + coord_flip() +

xlab("Exposure factors hyperuricemia ") +

ylab("Weight")

ggsave("tiff", plot = plot, width = 12, height = 8)

##Sensitivity analysis of the WQS model (train/validation split 7:3, random seed=2000)

WQS <- gwqs(HYPERURICEMIA~ wqs

+ BMI +Length of service+ Age+ Gender+ Smoking + Drinking,

mix_name = exposure,

data = data,

q = NULL,

validation = 0.7,

b = 1000,

b1_pos = TRUE,

b_constr = FALSE,

family = "binomial",

signal = "expt",

seed = 2000,

plots = TRUE,

tables = TRUE)

coef_original <- coef(WQS)

confint_original <- confint(WQS)

summary_WQS <- summary(WQS)

p_values <- summary_WQS$coefficients[, "Pr(>|z|)"]

result <- cbind(coef_original, confint_original, p_values)

colnames(result) <- c("Coefficient", "2.5% CI", "97.5% CI", "P-value")

print(result)

gwqs_weights_tab(WQS)

library(ggplot2)

gwqs_barplot(WQS)+

theme(panel.grid.major = element_blank(),

panel.grid.minor = element_blank(),

panel.background = element_blank(),

axis.line = element_line())

options(digits = 3)

gwqs_barplot(WQS)

w_ord <- order(WQS$final_weights$mean_weight)

mean_weight <- WQS$final_weights$mean_weight[w_ord]

mix_name <- factor(WQS$final_weights$mix_name[w_ord],

levels = WQS$final_weights$mix_name[w_ord])

dataplot <- data.frame(mean_weight, mix_name)

library(ggplot2)

plot <- ggplot(dataplot, aes(x = mix_name, y = mean_weight, fill = mix_name))+

geom_bar(stat = "identity", color = "black")+

geom_text(aes(label = sprintf("%.1f", mean_weight * 100)), hjust = -0.2, size = 4) +

theme_bw() +

theme(

axis.ticks = element_blank(),

axis.text.x = element_text(color = 'black', family = "Times New Roman", size = 14),

axis.text.y = element_text(family = "Times New Roman", size = 14),

axis.title.x = element_text(size = 16, family = "Times New Roman", margin = margin(t = 10)),

axis.title.y = element_text(size = 16, family = "Times New Roman", margin = margin(r = 10)),

legend.position = "none",

panel.grid = element_blank()

) +

scale_y_continuous(

labels = function(x) sprintf("%.0f%%", x * 100)

) + coord_flip() +

xlab("Exposure factors hyperuricemia ") +

ylab("Weight")

ggsave("tiff", plot = plot, width = 12, height = 8)

##Sensitivity analysis of the WQS model (train/validation split 5:5, random seed=2000)

WQS <- gwqs(HYPERURICEMIA~ wqs

+ BMI +Length of service+ Age+ Gender+ Smoking + Drinking,

mix_name = exposure,

data = data,

q = NULL,

validation = 0.5,

b = 1000,

b1_pos = TRUE,

b_constr = FALSE,

family = "binomial",

signal = "expt",

seed = 2000,

plots = TRUE,

tables = TRUE)

coef_original <- coef(WQS)

confint_original <- confint(WQS)

summary_WQS <- summary(WQS)

p_values <- summary_WQS$coefficients[, "Pr(>|z|)"]

result <- cbind(coef_original, confint_original, p_values)

colnames(result) <- c("Coefficient", "2.5% CI", "97.5% CI", "P-value")

print(result)

gwqs_weights_tab(WQS)

library(ggplot2)

gwqs_barplot(WQS)+

theme(panel.grid.major = element_blank(),

panel.grid.minor = element_blank(),

panel.background = element_blank(),

axis.line = element_line())

options(digits = 3)

gwqs_barplot(WQS)

w_ord <- order(WQS$final_weights$mean_weight)

mean_weight <- WQS$final_weights$mean_weight[w_ord]

mix_name <- factor(WQS$final_weights$mix_name[w_ord],

levels = WQS$final_weights$mix_name[w_ord])

dataplot <- data.frame(mean_weight, mix_name)

library(ggplot2)

plot <- ggplot(dataplot, aes(x = mix_name, y = mean_weight, fill = mix_name))+

geom_bar(stat = "identity", color = "black")+

geom_text(aes(label = sprintf("%.1f", mean_weight * 100)), hjust = -0.2, size = 4) +

theme_bw() +

theme(

axis.ticks = element_blank(),

axis.text.x = element_text(color = 'black', family = "Times New Roman", size = 14),

axis.text.y = element_text(family = "Times New Roman", size = 14),

axis.title.x = element_text(size = 16, family = "Times New Roman", margin = margin(t = 10)),

axis.title.y = element_text(size = 16, family = "Times New Roman", margin = margin(r = 10)),

legend.position = "none",

panel.grid = element_blank()

) +

scale_y_continuous(

labels = function(x) sprintf("%.0f%%", x * 100)

) + coord_flip() +

xlab("Exposure factors hyperuricemia ") +

ylab("Weight")

ggsave("tiff", plot = plot, width = 12, height = 8)
